# Food Vacuole Associated Enolase in *Plasmodium* Undergoes Multiple Post-Translational Modifications: Evidence for Atypical Ubiquitination

**DOI:** 10.1371/journal.pone.0072687

**Published:** 2013-08-23

**Authors:** Saudamini Shevade, Nitin Jindal, Sneha Dutta, Gotam K. Jarori

**Affiliations:** Department of Biological Sciences, Tata Institute of Fundamental Research, Colaba, Mumbai, India; University of Melbourne, Australia

## Abstract

*Plasmodium* enolase localizes to several sub-cellular compartments viz. cytosol, nucleus, cell membrane, food vacuole (FV) and cytoskeleton, without having any organelle targeting signal sequences. This enzyme has been shown to undergo multiple post-translational modifications (PTMs) giving rise to several variants that show organelle specific localization. It is likely that these PTMs may be responsible for its diverse distribution and moonlighting functions. While most variants have a MW of ~50 kDa and are likely to arise due to changes in pI, food vacuole (FV) associated enolase showed three forms with MW~50, 65 and 75 kDa. Evidence from immuno-precipitation and western analysis indicates that the 65 and 75 kDa forms of FV associated enolase are ubiquitinated. Using mass spectrometry (MS), definitive evidence is obtained for the nature of PTMs in FV associated variants of enolase. Results showed several modifications, viz. ubiquitination at K147, phosphorylation at Y148 and acetylation at K142 and K384. MS data also revealed the conjugation of three ubiquitin (Ub) molecules to enolase through K147. Trimeric ubiquitin has a linear peptide linkage between the NH_2_-terminal methionine of the first ubiquitin (Ub1) and the C-terminal G76 of the second (Ub2). Ub2 and third ubiquitin (Ub3) were linked through an atypical isopeptide linkage between K6 of Ub2 and G76 of Ub3, respectively. Further, the tri-ubiquitinated form was found to be largely associated with hemozoin while the 50 and 65 kDa forms were present in the NP-40 soluble fraction of FV. Mass spectrometry results also showed phosphorylation of S42 in the cytosolic enolase from *P. falciparum* and T337 in the cytoskeleton associated enolase from *P* *. yoelii* . The composition of food vacuolar proteome and likely interactors of enolase are also being reported.

## Introduction

Enolase is one of the most abundant glycolytic enzymes in the cytosol. It catalyzes the inter-conversion of 2-phosphoglycerate and phosphoenolpyruvate during glycolysis and gluconeogenesis, respectively. Apart from its cytosolic localization, the presence of this protein has also been reported on the plasma membrane [1–6], nucleus [7–11] and vacuole [5,12,13]. This diverse sub-cellular distribution of enolase is likely to be associated with a variety of different functions, e.g. serving as a plasminogen receptor on the cell surface [2,14], ligand for mosquito gut epithelial receptors [15,16], transcription factor in plants [8] and cancer cells [9], a component of RNA degradosome in *E. coli* [17], inhibitor of Dnmt2 in *Entamoeba histolytica* [7], structural component of eye lens [18], heat shock protein in yeast [19] etc. Like several other glycolytic enzymes, enolase also seems to be recruited for a variety of moonlighting functions in different organisms [1,20].

In *Plasmodium falciparum*, immuno-gold electron microscopic imaging to determine the sub-cellular distribution of enolase revealed its association with the food vacuole, in addition to it being present in the nucleus, cytosol and the cell surface [5]. Similar to enolases from several other organisms, parasite enolase also binds to plasminogen [5] and was suggested to have a role in invasion of RBCs [21]. Adaptive changes in the proteome of *P. falciparum* merozoites on switching the invasion dependence from sialated to non-sialated receptor on erythrocytes, showed up-regulation of enolase [22]. Another invasive stage of the parasite, ookinete that invades the mosquito gut wall has cell surface localized enolase. Enolase on the ookinete surface binds plasminogen as well as serves as a ligand for gut wall epithelial receptors. Blocking the surface localized enolase in ookinetes with anti-enolase antibodies prevented the invasion of gut epithelium [15,16]. Thus, two distinct cell surface functions for parasite enolase have emerged at ookinete stage, (i) to act as cell surface receptor for plasminogen and (ii) to act as ligand for mosquito gut wall epithelial receptors. Both these functional roles are important for the invasion of the mosquito gut wall by ookinete.

Attempts were made to obtain insights into the functional role of food vacuole associated enolase in *Plasmodium* [13]. Involvement of enolase in vacuolar fusion and protein trafficking to vacuole have been reported in yeast [12]. In yeast, enolase binds to a subunit of adaptor protein complex-3 [23,24], providing vesicle structure and cargo specificity for vesicles moving between the Golgi and the vacuole [25,26]. A recent observation about the association of enolase with tonoplast (plant vacuole membrane) and its probable interaction with v-ATPase has raised the possibility of the involvement of enolase in mediating salt tolerance in plants [27]. Although enolases are highly conserved across the species, evolutionarily, apicomplexan enolases are much more similar to those in plants as compared to other eukaryotes [28,29]. In the plant tonoplast, the molecular nature of enolase association with the vesicle membrane is not yet understood. In the absence of any transmembrane domain and/or vacuolar localization signal, it is likely that post-translational modifications in enolase may play a role in such functional associations. This hypothesis gets further credence from the observation that the amount of enolase associated with the plant tonoplast gets modulated by changes in ambient salt concentrations [27] suggesting the importance of signal transduction dependent PTMs.

In *Plasmodium* , food vacuole associated enolase showed three different variants with MW~50, 65 and 75 kDa. The relative amounts of the three isoforms exhibit variation from preparation to preparation, suggesting the possibility of inter-conversion among the three variants in response to environmental or developmental cues [13]. As a first step towards understanding this phenomenon and to elucidate the vacuolar functions of enolase in *Plasmodium* , we decided to (i) analyze the food vacuolar proteome and identify the protein homologs of enolase interactors known in vacuoles from other organisms (e.g. yeast) that are present in the parasite and (ii) determine the chemical nature of post-translational modifications in enolase associated with *Plasmodium* food vacuole.

## Materials and Methods

### Materials

Anti-recombinant *Plasmodium falciparum* enolase (rPfeno) antibodies were raised in house as described earlier [30]. Anti-mouse IgG (cat no. A5278) and anti-rabbit IgG (cat no. A8275) labeled with HRP and Percoll^TM^ were purchased from Sigma Chemical Co., St Louis, USA. Anti-ubiquitin antibody, 1B4-uB (cat no. ab 122) was obtained from Abcam. Anti-falcipain-2 antibody that was raised against amino acids 209-484 (in rabbit) was a gift from Prof. V. S. Chauhan, ICGEB, New Delhi, India. Anti-PfP0 (ribosomal P0 protein) was a gift from Prof. S. Sharma, TIFR, Mumbai, India. Western blotting substrate (Pierce product 32106) was supplied by Thermo Scientific. Sequencing grade trypsin was obtained from Roche Diagnostics. Protease inhibitor cocktail (100x solution) was purchased from Pierce (cat no. 78425), DNase I was obtained from Bangalore Genei. Protein A-Sepharose was obtained from GE Healthcare.

### Growing *Plasmodium* *yoelii*

8-10 weeks old Swiss mice were intra-peritoneally injected with ~10^6^ *P* *. yoelii* infected mouse RBCs. Parasitemia was monitored everyday with tail bleeding technique until it reached 30-40%. For the blood collection, animals were first anesthetized using isoflurane (to reduce the suffering) and then subjected to retro-orbital bleeding. Subsequently, animals were sacrificed by quick cervical dislocation and carcasses were stored at -20^o^C till incineration. Blood was collected in acid citrate dextrose (136 mM glucose, 41.6 mM citric acid and 74.8 mM sodium citrate). RBCs were collected by centrifugation and lysed using 0.05% saponin for the release of the intra-cellular parasite. Parasites were collected by centrifugation, snap frozen in liquid nitrogen and stored at -80^o^C as described earlier [13]. Experiments involving mice were approved by the Institutional Animal Ethics Committee (IAEC) of Tata Institute of Fundamental Research, formulated by the 'Committee for the Purpose of Supervision and Experiments on Animals (CPCSEA), Government of India (Project approval No: TIFR/IAE/2010-4 and TIFR/IAEC/2012-5).

### Parasite food vacuole (FV) preparation

FVs from *P* *. yoelii* were purified by the method of Saliba et al [31] as described earlier [13]. Briefly, ~100 mg of *P* *. yoelii* cells were suspended in 1 ml of ice-cold water [pH 4.5, containing protease inhibitor cocktail (1x)]. The cell suspension was subjected to trituration for 5 times with a 26.5 gauge needle. The triturate was centrifuged at 18000 x g using Eppendorf centrifuge-5810R for 2 minutes. The supernatant was discarded. The pellet was re-suspended in 1mL of ‘Uptake Buffer’ (uptake buffer contained 2 mM MgSO_4_, 100 mM KCl, 10 mM NaCl, 25 mM HEPES, 25 mM NaHCO_3_, and 5 mM sodium phosphate pH 7.4), to which 10 µL of 5 mg/mL of DNase I was added and incubated at 37^°^C for 5 minutes. This was again centrifuged at 18000 x g, and the supernatant was discarded. The pellet was re-suspended in 100 µL of ice cold Uptake Buffer and 1.3 mL ice cold 42% Percoll^TM^ with 0.25 M sucrose and 1.5 mM MgCl_2_, pH 7.4. This suspension was passed twice through a 26.5 gauge needle and centrifuged at 18000 x g at 4°C for ten minutes. The lowermost dark band on the gradient, which contained the FVs, was isolated, washed with Uptake buffer, and either frozen at -80°C or used immediately for experiments.

### Immuno-precipitation

For immuno-precipitation (IP) of enolase, whole cell soluble fraction and FV soluble fraction were prepared as follows. Parasite crude extract prepared by trituration of cells was centrifuged at 18000xg. Supernatant was collected and labeled as ‘Whole cell soluble fraction’. This fraction was free from all the particulate organelles (e.g. mitochondria, apicoplast, food vacuole, cytoskeletal components, cell membrane etc). The pellet was used for the purification of food vacuole. For pull down of enolase from the whole cell soluble fraction, it was incubated with the purified mouse anti-rPfeno IgGs and Protein A-Sepharose beads for 2 hours at 4^o^C on a rotary mixer. Beads were collected by centrifugation at 2000xg and washed with uptake buffer. Proteins bound on beads were analyzed on SDS-PAGE. For IP from FV preparation, the FV pellet was solubilized in 20 mM Tris-HCl pH 8 containing 137 mM NaCl, 1% NP-40 and 2 mM EDTA. This was subjected to centrifugation (18000 x g for 10 minutes at 4^o^C) and the supernatant (FV soluble fraction) was used for pull down experiments using the appropriate antibody. Pellet (FV insoluble fraction) that mostly contained hemozoin was analyzed by western for the presence of enolase, if any.

### Gel Electrophoresis, Western blotting and in-gel trypsin digestion

Samples were suspended in reducing gel sample buffer, treated at 95^o^C for 5 minutes and centrifuged at 16000 x g for 10 min. Supernatant was analyzed on a 10% SDS gel [32]. Western blotting was done as described earlier [13] and probed using anti-rPfeno (1:1000) and anti-ubiquitin (1:500) antibodies. Blots were incubated with the appropriate HRP labeled secondary antibodies for 45 minutes and washed. This was incubated with Pierce Western blotting substrate solution for ~1 minute and excess substrate was removed. The blot was exposed to Super Rx blue sensitive X-ray film (Fujifilm) and developed using Optimax 2010 X-ray film processor.

For the preparation of samples for mass spectrometric analysis, the gel was serially sliced, minced and digested with trypsin and peptides were extracted [33]. Briefly, minced gel pieces (~1 mm^3^) were washed with H_2_O (thrice) and destained with 100 mM ammonium bicarbonate: acetonitrile (ABC: ACN, 1:1). This was followed by reduction with 10 mM DTT at 55°C for 45 min and alkylation with 50 mM Iodoacetamide in dark at room temperature for 45 minutes. The gel pieces were washed thrice with 100 mM ABC: ACN (1:1) and then dehydrated using 100% ACN. Gel pieces were dried in vacuum evaporator. 20 µg trypsin in 100 µl of 1mM HCl was mixed with 900 µl of digestion buffer (40 mM ABC in 9% ACN). Gel pieces were covered with 20 µl (0.4 µg) trypsin, and kept on ice for gel pieces to absorb trypsin. Another 50 µl digestion buffer was added to cover the gel pieces. Digestion was carried out at 37°C for 16 hours. The supernatant was collected in fresh tubes. Peptides were extracted by sonication of the gel pieces in 50 µl of 50% ACN, 10% formic acid (FA) in a water bath for 20-30 minutes. Extraction was repeated thrice with increasing amount of ACN using 50%, 70% and 90% ACN in 10% FA. The supernatant was dried in a vacuum evaporator at 30°C and kept at -20°C till further use.

### LC-ESI-MS/MS Analysis

Peptides extracted from trypsin treated samples were analyzed by LC-ESI-MS/MS using an Agilent 6520-QTOF. Peptides were taken in 3 µl of 0.1% formic acid (FA) (Solvent A). Typically, 2 µl of this sample was applied to an Agilent HPLC chip (G4240-62002). Nano-chip comprised of a 40 nl enrichment column and a 75 µm x 150 mm separation column that was packed with Zorbax 300 SB-C18 (5 µm) material. After sample injection, the column was washed for 2 min with 0-3% Solvent B (90% ACN in 0.1% FA), and peptides were eluted for 2-7 min with 3-20%, 7-35 min with 20-45%, 35-45 min with 45-80%, 45-50 min with 80-100% Solvent B and held at 100% for 5 min more. Active exclusion was set-on for 0.5 min after each MS/MS spectrum. m/z range used was 50-3200. MS scan rate = 4 and MS/MS scan rate = 3 per min. For each MS, five most abundant precursor ions were sequenced.

The. mgf files so obtained were submitted for protein identification searches against ‘Alveolata database from NCBInr’ using an in house Mascot server. The parameters for the search were - carbamidomethyl (cysteine) was set as a fixed modification; oxidation of methionine, phospho (serine/threonine), phospho (tyrosine), acetyl (lysine) and glygly (lysine) were set as variable modifications. Two missed cleavages were allowed. Peptide mass tolerance was set to 10 ppm and MS/MS mass tolerance 0.6 Da. Peptide charges were set to 2^+^, 3^+^ and 4^+^.

## Results

### *P. yoelii* FV proteome

For the identification of proteins present in food vacuole, purified preparation of *P* *. yoelii* food vacuole was analyzed on a SDS-gel and the gel lane was cut into 14 slices. Each slice was chopped in to smaller pieces and digested with trypsin. Extracted peptides were subjected to MS/MS analysis for the identification of proteins and post-translational modifications. Table S1 lists the proteins that were identified in the vacuolar preparation. In all, 298 proteins were detected. Some of these identified proteins were either involved in vesicular trafficking or are components of the mature food vacuole. Several others are likely to be minor contaminants arising from other organelles. Table S2 lists the previously known vacuolar proteins (or their homologs) among these 298 proteins. These proteins are likely to be either involved in vesicular trafficking or constitute resident components of the food vacuole. Interestingly, we could identify enolase in a gel band around a molecular mass of ~65-80 kDa. This was in agreement with our earlier observation of enolase using western blot analysis [13]. In a recent report, several proteasomal proteins have been shown to be associated with the yeast vacuole [34]. Several proteasomal proteins were also found to be associated with food vacuole. Possibility of the presence of such proteasomal proteins in association with parasite food vacuole was examined by setting up a search for yeast proteasomal protein homologs in the FV proteome. Some of the proteins identified in the food vacuole proteome are also listed in Table S2 as possible components of the FV. However, presence of proteasomal components in FV proteome can also arise due to proteasomal contamination of FV preparations. Such a possibility cannot be ruled out. In an earlier study, similar proteome analysis on *P. falciparum* FV preparation yielded >116 proteins [35]. However, these authors failed to detect enolase in FV preparation. In general, analysis of organellar proteomes by mass spectrometry tends to identify a large number of minor contaminants that are usually not observed in western analysis [13,35,36]. This is likely to be due to high sensitivity of detection in MS experiments.

### FV associated *Plasmodium* enolase and post-translational modifications

In an earlier study, two high molecular mass variants (~65 and 75 kDa) of enolase were detected in food vacuole proteome analyzed on SDS-PAGE and visualized using Western blot. The 65 kDa variant appeared as if it is an intermediate to the formation of ~75 kDa variant [13] as sometimes it was absent in the vacuolar proteome. Presence of such variants suggested the possibility of conjugation of the ~50 kDa native enolase with other proteins. One such candidate could be ubiquitin that has a mass of ~8 kDa. Attachment of two and three molecules of ubiquitin to enolase can account for observed variants that are ~15 and ~25 kDa greater in mass than the native enolase. These possibilities were tested using Western blot analysis as described below.

### FV associated enolase may be ubiquitinated

**Figure 1:**
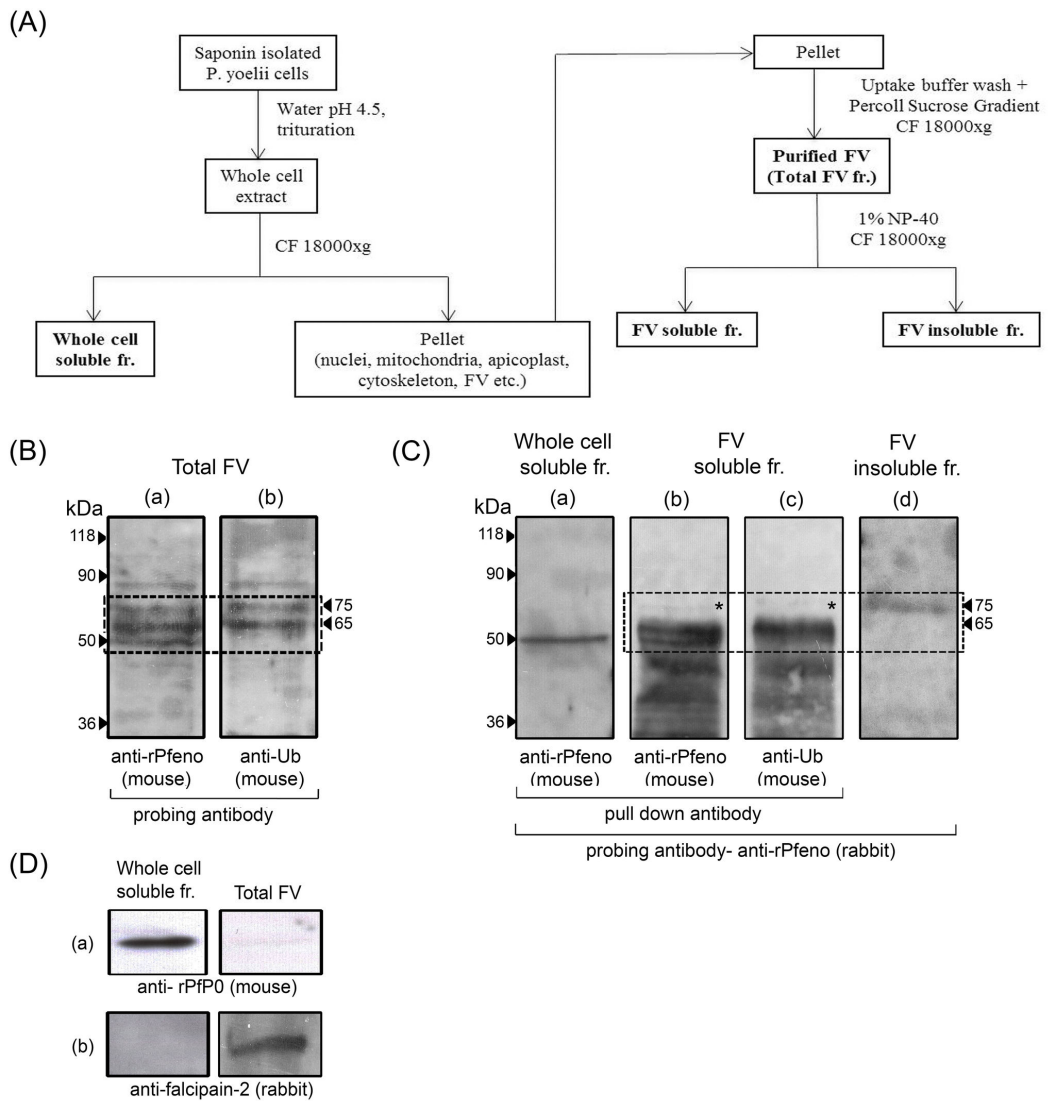
Analysis of *P* *. yoelii* food vacuole (FV) associated enolase variants using antibody pull down assays and western blots. (A) *P* *. yoelii* cell fractionation scheme. Various fractions used for analysis are marked in bold. Proteins were separated on 10% SDS PAGE. (B) Western blot of FV proteome probed with (a) anti-rPfeno (mouse) and anti-Ub (mouse) antibodies. Note that three variants of enolase were observed while two of these (65 & 75 kDa) are ubiquitinated (dotted line box). Matched amounts of proteins were loaded in both the lanes. (C) Antibody pull-down assays for different sub-cellular fractions using anti-rPfeno (mouse) and anti-Ub antibodies (mouse). Blots of pull-down proteins were probed with anti-rPfeno (rabbit) to detect the presence of enolase. (D) Western blots of whole cell soluble fraction and total FV probed with ant-PfP0 (cytosolic marker) and anti-falcipain-2 (FV marker) antibodies. Equivalent amount of total proteins were loaded in each lane. Note the enrichment of falcipain-2 and near absence of P0 in FV preparation indicating that preparation used in above experiments is highly enriched in FV.

To test the possibility of FV associated enolase being ubiquitinated, parasite cells were fractionated as shown in Figure 1A. Some of the relevant fractions were subjected to antibody based pull down assays and western analysis. These are marked in bold in Figure 1A. To examine the possibility of ubiquitination of FV associated enolase, FV pellet was dissolved in SDS-PAGE sample buffer and analyzed on a 10% gel. Equivalent amounts of the sample were run in two different lanes and gel was subjected to western analysis using mouse anti-rPfeno and anti-ubiquitin antibodies, respectively. Results are shown in Figure 1B. Similar to our earlier results [13], the first gel lane that was probed with r-Pfeno antibody showed three variants of enolase with MW~50, 65 and 75 kDa respectively [Figure 1B(a)]. The second lane that was probed with anti-Ub antibody showed reactivity with the two high molecular mass variants with MW~65 and 75 kDa [Figure 1B(b)] (these bands are enclosed in a dotted box in Figure 1B). Both these lanes also showed reactivity with a protein band about 90 kDa. However, this band was not observed in the antibody pull down assays [Figure 1C (b), (c) & (d)] and is likely to be a non-specific interaction. These results suggest that the two high molecular mass enolase positive bands (i.e. 65 and 75 kDa) associated with FV might arise due to conjugation of enolase with ubiquitin.

### Enolase is present in the ubiquitinated fraction of FV proteome

To determine whether the FV associated enolase is indeed ubiquitinated, a preparation of food vacuole was solubilized in a buffer (20 mM Tris-HCl pH 8, 137 mM NaCl, 2 mM EDTA) containing 1% NP-40. The extract was centrifuged and fractionated in 18000xg supernatant (FV soluble fraction) and the pellet (FV insoluble fraction). FV soluble fraction was split in two parts and used for immuno-precipitation. To the first aliquot, purified anti-rPfeno IgGs (mouse) and to the second aliquot anti-Ub (mouse) antibodies were added along with Protein A-Sepharose beads. After incubation, beads were collected by centrifugation. Proteins associated with the two pull down samples representing enolase variants and ubiquitinated fraction of FV proteome respectively, were analyzed on SDS-PAGE and subjected to western analysis using anti-rPfeno antibodies (rabbit). As expected, anti-rPfeno pull down sample showed three enolase bands [Figure 1C(b)] while anti-Ub pull down had two bands [Figure 1C(c)] corresponding to two higher molecular mass forms of enolase as observed earlier [Figure 1B(b)]. Ability of anti-Ub antibodies to pull down 65 and 75 kDa variants of enolase from the soluble FV fraction conclusively indicates that these two forms of enolase arose due to ubiquitination. In the FV soluble fraction, only trace amounts of 75 kDa variant (indicated by *) was observed as most of it fractionated with the FV insoluble fraction [Figure 1C(d)]. A pull down from whole cell soluble fraction (free from FV) using anti-rPfeno antibody had only 50 kDa variant [Figure 1C(a)] supporting the view that high molecular mass variants are associated with the food vacuole fraction. Low molecular bands observed in Figure 1C (b) & (c) arose due to proteolysis. As FV preparations are rich in proteolytic enzymes, solubilization of total FV fractions followed by prolonged incubation for pull down protocols resulted in partial proteolysis.

In order to rule out the possibility of any significant cytosolic contamination of FV preparation, equivalent amounts of protein from whole cell soluble fraction and total FV fractions were subjected to western analysis for the presence of a cytosolic marker protein PfP0 (ribosomal protein P0). The results showed that while whole cell soluble fraction has this protein, FV preparation is almost free from it [Figure 1D(a)]. As a positive control, both the fractions (soluble whole cell fraction and Total FV) were also analyzed for the presence of falcipain-2 (a proteolytic enzyme present in FV). Western blots showed absence of falcipain-2 in whole cell soluble fraction while it got enriched in Total FV fraction [Figure 1D(b)]. Absence of P0 protein and enrichment of falcipain-2 in the purified FV indicate high purity of the preparation used here.

### The 75 kDa variant of enolase is associated with hemozoin

The pellet from NP-40 solubilized FV preparation largely consists of hemozoin. Hemozoin associated proteins were extracted by treating the pellet with SDS gel sample buffer and collecting the supernatant by centrifugation. Supernatant was analyzed on a SDS gel and subjected to Western blot analysis to detect the presence of enolase. The ~75 kDa variant of enolase was found to be present in hemozoin pellet fraction [Figure 1C(d)]. Partial solubilization of this form of enolase was observed in some samples of NP-40 solubilized FV [see * in Figure 1C(b & c)]. These results support the view that the ~75 kDa form of FV associated enolase is bound to hemozoin while 50 and 65 kDa forms are not. Although these results do not rule out the possibility of non-specific binding of ~75 kDa variants with hemozoin, the selective association raises the possibility of some functional role for this form in hemozoin formation. However, such a possibility requires more definitive experimental data. Thus, the antibody pull down and western analysis experiments on parasite sub-cellular fractions described above, showed that the two high molecular wt variants of enolase associated with FV arose due to ubiquitination of native 50 kDa form and 75 kDa variant selectively binds to hemozoin.

### Mass spectrometric analysis of FV associated enolase showed ubiquitination

**Figure 2:**
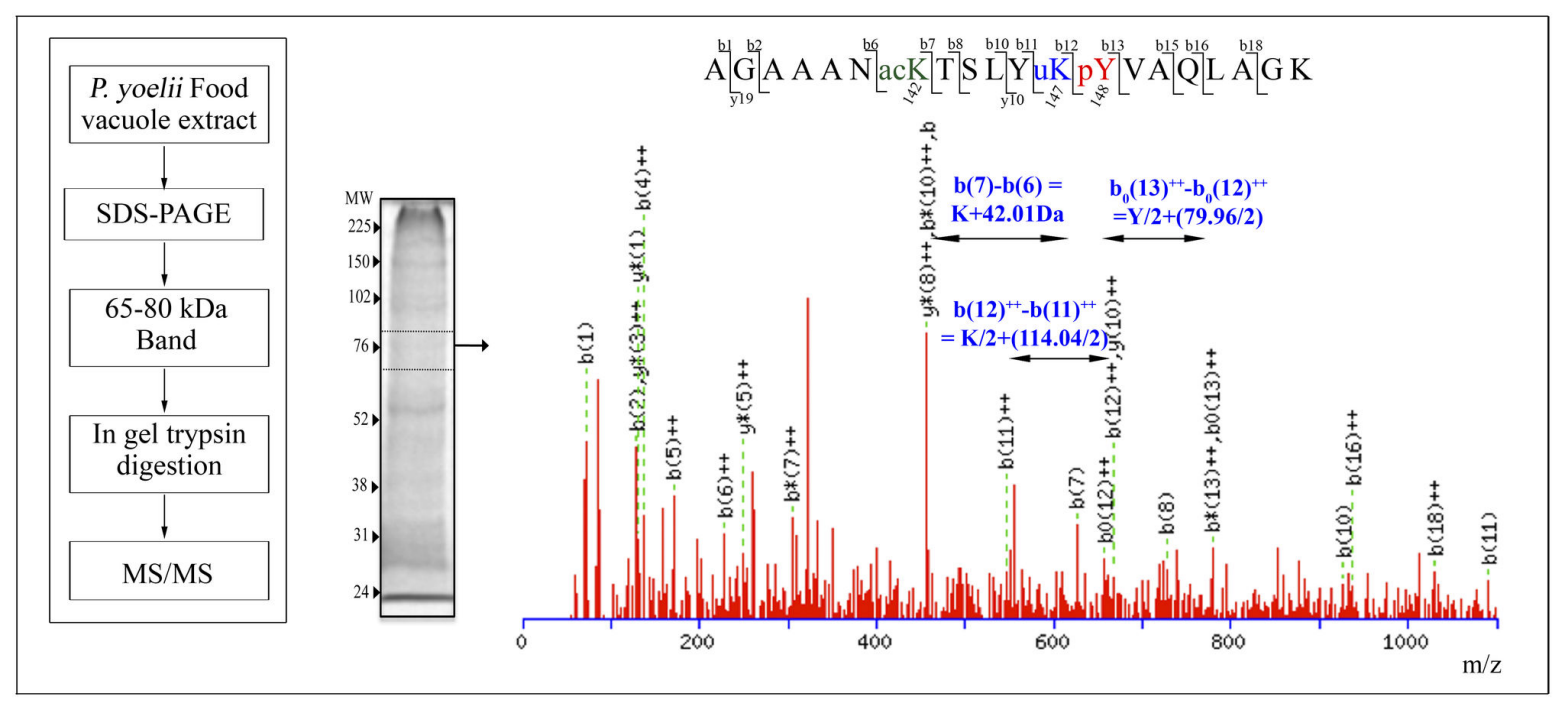
MS/MS spectrum of a peptide from *P* *. yoelii* enolase (Pyeno). Sequence covers residues 136-155 of Pyeno. Three different post-translational modifications are observed. K142 is acetylated, K147 has attached glycylglycyl residues through a trans-peptide linkage (a signature of ubiquitination) and Y148 is phosphorylated. ‘b’ ions corresponding to relevant modifications are marked with arrows along with aminoacyl residue mass + Δm for the modifying group.

In order to obtain definitive evidence for the ubiquitination of enolase and identify the site(s) at which such post-translational modifications had occurred, proteins from a food vacuole preparation were resolved on a SDS-PAGE and a ~65-80 kDa band (that is likely to have high molecular mass variants of Pyeno) was sliced out, digested with trypsin and subjected to MS analysis. MS/MS experiments showed several peptides from Pyeno that could be sequenced. Sequence coverage of ~73% was obtained for enolase with a high MWSE score of 279. These results were in agreement with the earlier data from immuno-electron microscopy (IEM) and western blotting where association of enolase with FV was first reported [5,13]. Additional information about the post-translational modifications in enolase could be deduced by detailed analysis of MS/MS data collected on the sample. Figure 2 shows an MS/MS spectrum of a peptide from Pyeno. This spectrum arose from the peptide consisting of residues 136-155 of Pyeno. The sequence showed three different post-translational modifications in this peptide, viz. acetylation at K142, ubiquitination at K147 (as evident by presence of –GG residues that increases the mass of K by 114 Da) and phosphorylation at Y148. Another modification that was identified in FV associated enolase was acetylation at K384.

MS/MS spectrum for the peptide^366^ VNQIGSITEAIEACLLSQ**K**NNWGVMVSHR^394^, containing residue K384 is shown in Figure S1.

### Ubiquitin derived peptides could be identified in the ~65-80 kDa gel band from FV proteome

**Figure 3:**
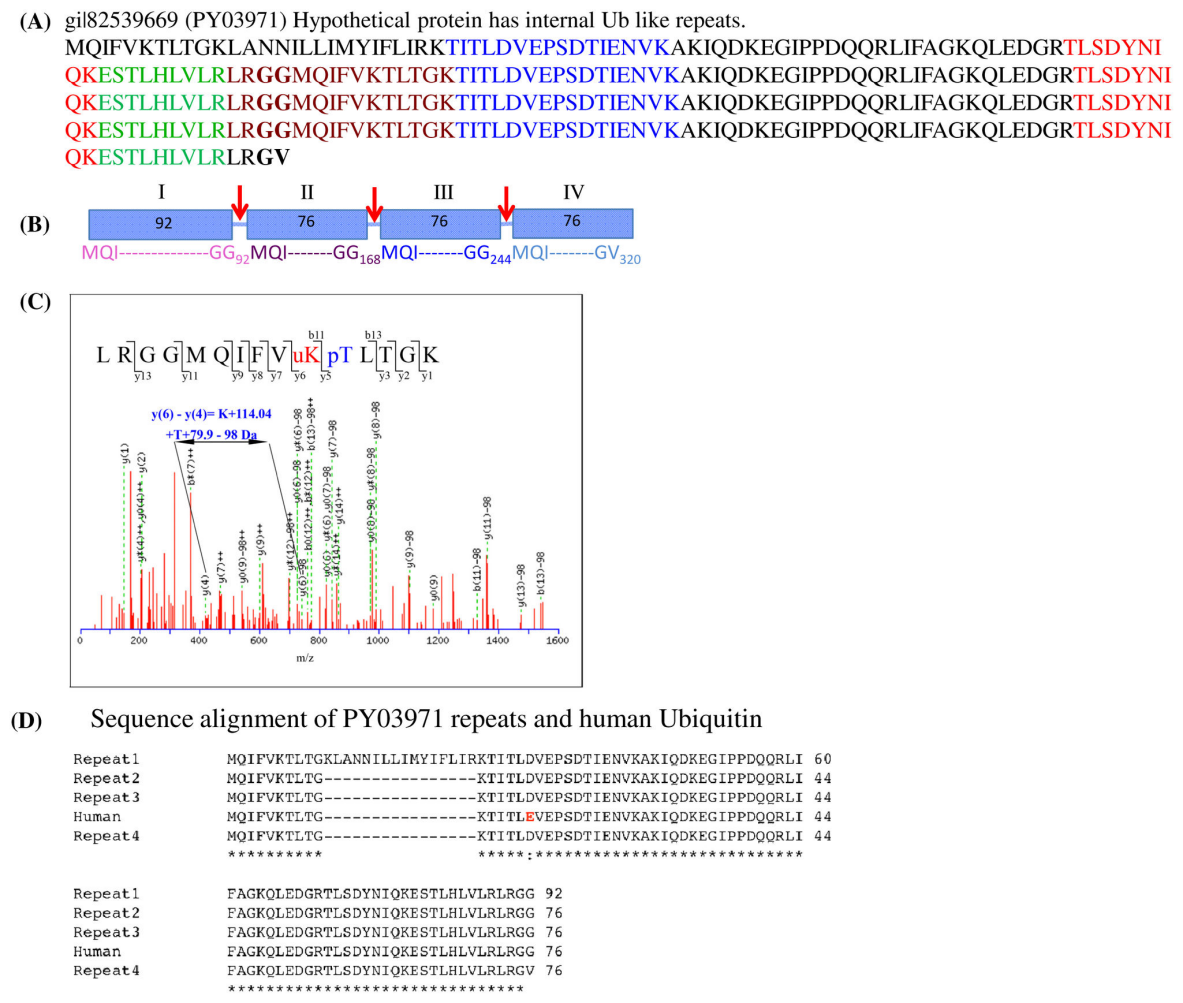
Ubiquitin moiety in *P* *. yoelii* . (A) *P* *yoelii* hypothetical protein (NCBI id: gi| 82539669; or Plasmodb id: PY03971 hypothetical protein) has four ubiquitin like internal repeats. Residues in blue, red, green and magenta belongs to peptides that were identified in MS analysis of tryptic digest of ~65-80 kDa band from FV gel. (B) Schematic representation of four ubiquitin repeats with cleavage sites shown in red arrows. Note that first subunit has an insert of 16 residues (aa11-26) raising its MW to ~92 kDa. Last repeat C-terminus has –GV instead of a –GG. (C) MS/MS spectrum of a peptide fromputative ubiquitin of *P* *. yoelii* putative ubiquitin. This peptide was present in the tryptic digest of the food vacuole SDS-PAGE 65-80 kDa band (see Figure 1). The peptide showed a linear peptide linkage between –G76 and M1 of two µb (Ub1 and Ub2) molecules while the K6 of Ub2 is the site for attachment of third Ub (Ub3) molecule. (D) CLUSTAL W alignment of four repeats from PY03971 protein and human ubiquitin. Repeats 2 and 3 have the same sequence as that of human (and mouse) except E16 in human (and mouse) is replaced with a D in *Plasmodium* *spp* . Observation of ‘D16’ in sequenced peptides [see (A)] indicates the Ub linked to protein in ~65-80 kDa band is of parasite origin and not that of host.

If higher molecular mass variants of *Plasmodium* enolase arose by ubiquitination, it is expected that the peptides derived from the trypsin digest of proteins in ~65-80 kDa gel band of FV proteome should contain peptides originating from ubiquitin. A search based on MS data in NCBI *P* *. yoelii* database led to the identification of gi|82539669 (PlasmoDB gene ID: PY03971), a hypothetical protein containing four repeats of ubiquitin sequence. Four different peptides, viz -TITLDVEPSDTIENVK-, TLSDYNIQK-, -ESTLHLVLR- and –LRGGMQIFVKTLTGK- belonging to this hypothetical protein got sequenced. Figure 3(A) shows the complete sequence of this protein where four sequenced peptides are marked in blue, red, green and magenta, respectively. The total sequence coverage obtained in MS sequencing was ~65% with MWSE score of 39. The MS/MS spectra for various peptides are shown in Figure 3(C) and Figure S2. PY03971 has four internal repeats and proteolytic processing can yield four fragments, of which two have the sequence of ubiquitin [Figure 3(B) II and III] while fragment-I has a 16 amino acid insert and fragment-IV has –GV at C-terminal (in place of –GG in ubiquitin). It is quite likely that fragment-I that has a large insert and the fragment-IV that has –GV at C-terminal, may not serve as substrates for ubiquitin conjugating enzymes (E1, E2 and E3), leaving the remaining two fragments (II and III) of PY03971 to be used for protein ubiquitination. Sequence alignment with human (or mouse) ubiquitin showed a conservative replacement of E16 in human ubiquitin with a ‘D16’ in the parasite (Figure 3D). One of the sequenced peptides from PY03971, -TITL**D**VESDTTIENVK- has aspartate (in place of glutamate in mice as shown in bold) indicating that the sequenced ubiquitin is of parasite origin and not from the host (mouse) where the parasites were cultured. Detection of ubiquitin (76 aa; MW~8 kDa) in the high molecular mass gel band of the FV proteome indicates the presence of ubiquitinated protein(s) in the FV. In the gel band (~65-85 kDa) analyzed here, about ~100 different proteins could be identified. However, none of the peptides derived from these proteins showed ubiquitination signature residues (i.e. diglycine attached to K) attached as PTM except the Pyeno (Figure 2). The conclusion that the two high molecular mass bands of enolase observed in FV proteome arose due to ubiquitination of Pyeno is evident from the following facts: (i) the ~65 & 75 kDa protein bands reacted with anti-enolase and anti-ubiquitin antibodies (Figure 1B), (ii) anti-Pfeno and anti-Ub antibodies are able to pull down both (~65 & 75 kDa) bands from NP-40 solubilized food vacuoles (Figure 1C), (iii) mass spectrometric detection of enolase and ubiquitin peptides in tryptic digest of proteins from ~65–80 kDa band (Figures 2 & 3); and (iv) the presence of -GG (signature of ubiquitination) on K147 in *Plasmodium* *yoelii* enolase (Pyeno) (Figure 2). Conjugation of two and three ubiquitin molecules to 50 kDa Pyeno can give rise to ~65 and ~75 kDa forms that are associated with *Plasmodium* food vacuoles [13].

### Nature of inter ubiquitin linkages and structure of tri-ubiquitinated Pyeno

**Figure 4:**
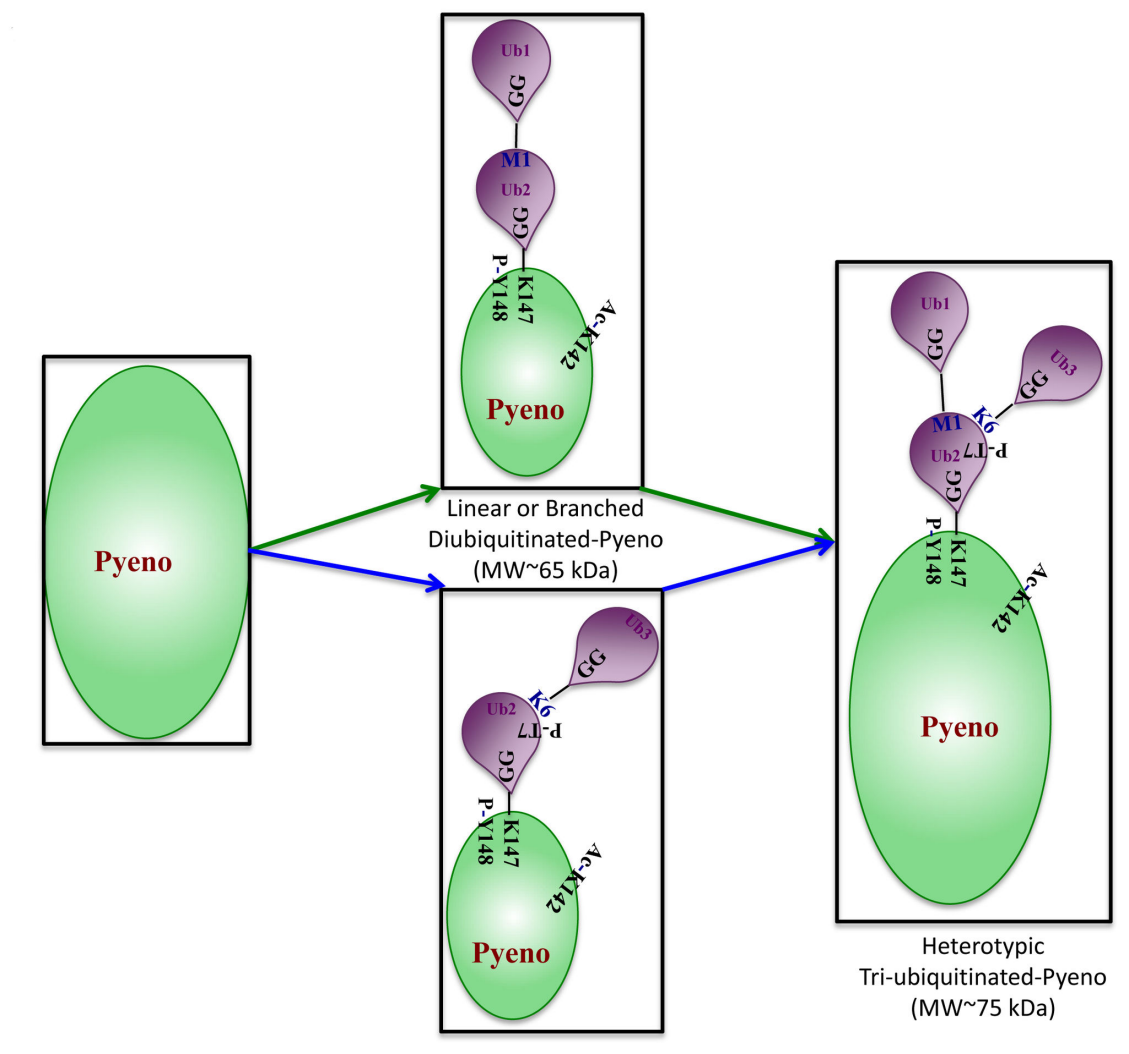
Schematic representation of post-translational modifications in Pyeno (determined in this study). It also shows two different intermediates with MW~65 kDa, that are possible on diubiquitination of Pyeno.

The evidence for the nature of linkages and number of ubiquitins conjugated with Pyeno came from the sequencing of a peptide that originated from conjugated ubiquitins. Figure 3(C) shows an MS/MS spectrum of a peptide from the tryptic digest of ~65-80 kDa gel slice that yielded a peptide sequence –LRGG*MQIFVuKpTLTGK-. This peptide has two PTMs, (i) ubiquitination signature of –GG at K6 (shown as uK) and (ii) phosphorylation of T7 (shown as pT). This sequence arises due to formation of a linear dimer by the attachment of the C-terminal –LRGG of ubiquitin one (Ub1) with N-terminal M1 of ubiquitin two (Ub2). This peptide linkage is shown with an asterisk (*) symbol. K6 of Ub2 bears a –GG signature (attached through an isopeptide linkage) where third ubiquitin (Ub3) molecule is linked with its C-terminal G76. Thus Ub2 forms a linear peptide linkage with Ub1 and a branched chain isopeptide linkage at K6 with Ub3. Covalent attachment of dimer (linear Ub1-Ub2 or branched Ub2-Ub3) or trimeric forms of ubiquitin to Pyeno can account for the formation of the ~65 and 75 kDa variants detected in Western blot analysis of FV associated Pyeno [13]. An attempt was made to model the tri-ubiquitinated Pyeno. The linear peptide linkage between Ub1 and Ub2 has an ‘Open’ conformation while the K6 branched inter-ubiquitin linkage between Ub2 and Ub3 is in a ‘Closed’ conformation. Such differences may form the structural basis for differential recruitment of two possible di-ubiquitinated forms and the trimeric form for varied physiological functions. Figure 4 shows a schematic diagram of all the PTMs in FV associated Pyeno and likely structures of di and tri-ubiquitinated Pyeno variants. Formation of linear ubiquitin dimer by ‘head to tail’ joining as well as branched structures generated through isopeptide linkages involving different lysine residues, give rise to heterotypic polyubiquitinated proteins. Such atypical structures have been reported earlier. However, their role in regulating physiological processes, remain vaguely understood [37–39].

### Phosphorylations in *Plasmodium* *spp.* enolase

Since several electrophoretic variants of enolase in *Plasmodium* *spp* have been reported [11,40,41] and some of these were predicted to arise due to phosphorylation, effort was made to identify the modified residue(s) in such variants. The soluble fraction from whole cell extract of *P. falciparum* was analyzed on SDS-PAGE and enolase containing gel band (MW~50 kDa) was sliced, trypsin digested and peptides were analyzed on mass spectrometer. MS/MS spectra of an enolase peptide covering sequence from 35–58 residues showed phosphorylation at S42 (Figure S3). Similarly, enolase containing gel band from cytoskeletal fraction derived from *P* *. yoelii* cell extract showed phosphorylation at T337. MS/MS spectra from phosphorylated and native form of these peptides covering residues 323-338 are shown in Figure S4. All the PTMs identified in *Plasmodium* enolase are listed in Table 1. Residues with post-translational modifications were also mapped on modeled 3D-structures of parasite enolases (Figure S5).

**Table 1:** Post-Translational modifications in *Plasmodium* *spp* Enolase.

**Gel Band MW (kDa)**	**Sub-cellular Fraction**	Peptide Sequenced*	**Modification**	**Residue number**	**Species**	**Peptide Score**	**Enolase Score**	**Enolase % Sequence Coverage**
~65-80	Food vacuole	AGAAAN**K**TSLYKYVAQLAGK	acetylation	K142	*P* *. yoelii*	8	279	71
~65-80	Food vacuole	AGAAANKTSLY**K**YVAQLAGK	ubiquitination	K147	*P* *. yoelii*	8	279	71
~65-80	Food vacuole	AGAAANKTSLYK**Y**VAQLAGK	phosphorylation	Y148	*P* *. yoelii*	8	279	71
~50	Food vacuole	VNQIGSITEAIEACLLSQ**K**NNWGVMVSHR	acetylation	K384	*P* *. yoelii*	18	467	58
~50	Cytoskeleton	DVQIVGDDLLVTNP**T**R	phosphorylation	T337	*P* *. yoelii*	19	416	44
~50	Cytosolic	AAVPSGA**S**TGIYEALELRDNDKSR	phosphorylation	S42	*P. falciparum*	28	2099	62

Various sub-cellular fractions were run on SDS-Page and enolase containing gel bands were subjected to MS/MS analysis for the determination of post-translational modifications.

* Modified residues are marked in bold.

## Discussion

*Plasmodium* food vacuole is a lysosome-like compartment with complex physiological functions essential for the survival of the organism. The food vacuole is involved in the proteolytic processing of massive amounts of hemoglobin to provide amino acids essential for the growth and rapid multiplication of the parasite in intra-erythrocytic stages. It also detoxifies heme by converting it into hemozoin. Proteins like HDP (heme detoxification protein) are believed to be involved in such conversion [42]. Interruption of vacuolar processes invariably leads to inhibition of parasite growth and multiplication. This has made molecular components of FV attractive targets for chemotherapeutics against malaria [43]. Analysis of food vacuole proteome and identification of putative orthologs of vacuole-associated proteins (Table S2) provide more candidates whose essentiality of function in parasite growth/multiplication can be evaluated. This will pave the way for their validation as newer drug targets.

Observation of the sub-cellular distribution of enolase in the parasite cell using immuno-electron microscopic (IEM) imaging, showed its association with food vacuole at different stages in the parasite life cycle [5]. Interestingly, there appear to be two different locations in FV where enolase is localized, (i) with the vacuolar membrane and (ii) with hemozoin in early trophozoite stages which gradually diminishes in late trophozoite-mid schizont stages [5]. It is interesting to compare the localization of enolase in FV with the published IEM images of MSP1_19_ [44] and HDP [42]. All three proteins, i.e. enolase, HDP and MSP1_19_ exhibit very similar pattern of association with hemozoin in food vacuole.

HDP is known to be involved in conversion of heme to hemozoin. It makes its way to the FV through an “outbound-inbound” trafficking route [42]. However, MSP1_19_, a proteolytically cleaved product of MSP1 (present in abundance on merozoite surface), gets endocytosed into small vacuoles that coalesce to form food vacuole containing hemozoin. MSP1_19_ is believed to play a significant role in the biogenesis and function of food vacuole [44]. Similarly, the presence of enolase on merozoite surface and its possible involvement in RBC invasion has been suggested [5,21]. MSP1 and Pfeno also showed co-localization on cell surface of a mature schizont (Figure S6). Thus, it is possible for enolase to reach FV through the pathway similar to MSP1_19_.

Alternatively, a small fraction of cytosolic enolase can bind to endocytic vesicles assisting in their fusion (as in the case of yeast [12]) and in turn get incorporated into the FV. This suggestion is also supported by the observed complementation of vacuolar fragmentation phenotype in enolase deficient yeast by parasite enolase [13]. In the enolase deficient yeast, several proteins (viz Sec18p, Vam3p, Nyv1p, Vti1p, Vam6p, Vps33p etc.) involved in vacuolar fusion have lesser amounts associated with vacuoles as compared to the wild type [12]. Later on it was suggested that in yeast, enolase might form a complex with Apl6 [23,24]. Apl6 is a component of AP3 complex, which provides an alternate route for transporting proteins to vacuoles that bypasses multi-vesicular body [25,26,45]. The possibility of similar pathway being functional in *Plasmodium falciparum* was supported by the fact that each of the AP3 complex components in yeast, had a putative ortholog in the parasite [13]. One can ask the question that in the food vacuole proteome of *P* *. yoelii* (Table S2), how many putative orthologs of the AP3 complex components are present? Table 2 shows a list of yeast proteins (AP3 components) and corresponding orthologs identified by genome wide search. *P* *. yoelii* FV proteome showed the presence of orthologs of Sec18, Ypt6, Vac8 and Eno1/2. Interactors of enolase identified in Y2H analysis in *P. falciparum* had identified three interactors, namely Vam6, Sec31 and Vac8 [46]. Thus several molecular components (orthologs) of AP3 complex are associated with food vacuole of *Plasmodium* (Table 2). However, direct interaction between enolase and other components of AP3 complex is yet to be demonstrated.

**Table 2:** Interactome of vacuolar enolase in yeast, *P. falciparum* and *P* *. yoelii* .

**Sr. No.**	**Yeast**	***P. falciparum* orthologs**	** *P* *. yoelii* orthologs**
1	SEC 18	PFC0140c N-ethylmaleimide-sensitive fusion protein, putative	**PY05628 (NCBI:gi|82596078) ATPase, AAA family**
2	VPS33	PFI1700c vesicle transport protein, putative	PY01196, vacuolar protein sorting homolog r-vps33a
3	***VAM6***	***PFL1340chypotheticalprotein,conserved***	PY00537 hypothetical protein
4	VTI1	PFL1740w, hypothetical protein, conserved	PY04228 hypothetical protein
5	VAM3	PFL2070w, t-SNARE, putative	PY03571 hypothetical protein
6	NYV1	MAL13P1.16, synaptobrevin-like protein, putative	PY03903, At5g22360/MWD9_16, putative
7	BET1	PF10_0109, conserved *Plasmodium* protein, unknown function	No ortholog found in P. yoelii
8	***SEC31***	***PFB0640c,sec31pputative***	PY03930 hypothetical protein
9	YPT6	PF11_0461, PfRab6, GTPase	**PY00721 (NCBI:gi|82596931) hypothetical protein**
10	***VAC8***	***MAL13P1.308,conserved* *Plasmodium* *protein* *,unknownfunction***	**PY01759 (NCBI:gi|83273805) hypothetical protein ( *P* *. yoelii* 17XNL)**
11	APL6	PFF0655c, adapter-related protein, putative	PY01672 adapter-related protein complex 3 beta 2 subunit
12	***ENO1/2***	***PF10.0155,Enolase***	**PY06644 (NCBI:gi|82752500) Enolase**

Enolase interactome for the yeast vacuole was earlier inferred from Saccharomyces genome database (SGD) and other studies [13]. Orthologs in *P. falciparum* and *P* *. yoelii* inferred from blast searches are listed below. Enolase interactors identified in *P. falciparum* using Y2H are shown in bold italics [46]. *P* *. yoelii* FV proteome analysis reported here identified three interactors (shown in bold).

There are three different variants of enolase (50, 65 and 75 kDa forms) that are associated with food vacuole. NP-40 solubilization of FV showed that 50 and 65 kDa forms could be extracted in detergent solubilized fraction [Figures 1C(b) and 1C(c)] while 75 kDa form remained largely in association with hemozoin [Figure 1C(d)]. It is tempting to speculate that the low mass variant of enolase (~50 kDa) that is likely to be associated with the food vacuolar membrane, may be involved in vacuolar fusion process while the high molecular mass variant (75 kDa) may perform heme detoxification related functions inside the vacuole. Thus, it is being suggested that different variants of enolase present in FV may have different functional roles. However, in the absence of supporting experimental evidence, this remains to be demonstrated. Another glycolytic enzyme that has been implicated to be involved in vesicular processes in *Plasmodium* is glyceraldehyde 3-phosphate dehydrogenase [47,48]. However, exact molecular mechanisms for their vacuolar function(s) are not yet understood.

Post-translational modifications (PTMs) of proteins are common mechanisms for controlling signal transduction, protein–protein interaction and sub-cellular localization. This is also nature’s way to expand its repertoire of primary gene products to provide many more molecular components [49]. There are three different isozymes of enolase in human beings, ENO1, ENO2 and ENO3 that have tissue specific expression. ENO1 that is expressed in most tissues is known to undergo a multitude of post-translational modifications in normal and tumor tissues, some of which are different in normal and diseased states and may have value in therapeutic and diagnostic strategies [50]. Comparison of residues that undergo modifications in Pyeno and human ENO1 (Figure S7) showed that except an active site serine, all other modified residues in parasite enolase are not conserved in human ENO1. Among the multiple PTMs reported for enolases, so far ubiquitination has not been found. Thus, this is the first report finding tri-ubiquitination of FV associated enolase in *Plasmodium* . Such information about differential modifications in host and parasite proteins can be of potential use in new therapeutics. Tri-ubiquitin moiety attached to Pyeno has two different types of linkages, (i) linear linkage between G76 of Ub1 and M1 of Ub2 and (ii) K6 of Ub2 and G76 of Ub3. This resulted in branched mixed-linkage chain attached to Pyeno. Different types of linkages impart different specific conformations to the coupled ubiquitins (e.g. linearly linked ubiquitins have open conformation while the two K6 linked moieties are in closed conformation) and are likely to be recognized by different sets of molecular machineries associated with different functions. For example, monoubiquitination has been shown to control processes such as DNA repair and signaling function in endocytic pathway [51], homotypic polyubiquitination using K48 linked chain serves as a signal for proteasomal degradation [52] whereas the K63 linked polyubiquitin chain functions in signal transduction and DNA repair [53]. Attempts to correlate the type of linkage with physiological function had reasonable success in cases where all the linkages were of single type (homotypic) [54]. Heterotypic linkages did not conform to this model. Linear and K6 linked trimeric ubiquitin conjugated to Pyeno is structurally distinct from several other characterized chains, possibly hinting at a different biological function that this linkage may regulate [55].

MS/MS spectrum of Pyeno peptide (Figure 2) provides evidence for the phosphorylation of a tyrosine residue (Y148). Detection of pY suggests the presence of tyrosine kinase activity that may be either of the parasite or of the host origin. Computational analysis of genome sequences has retrieved ~66-99 protein kinases. However, none of these belonged to tyrosine kinase (Tyrk) family [56–58]. As Tyrks are known to function in pathways essential for inter-cellular communication, it was surmised that these might not be needed in unicellular parasites like *Plasmodium* . In a recent study on phosphoproteome of *P. falciparum* and *T. gondii*, several tyrosine phosphorylated proteins have been identified [59], raising the possibility that these parasites may have novel class of tyrosine kinases. Observation of phosphorylation of Y148 (Figure 2) and T7 (Figure 3(C)) residues in the vicinity of ubiquitination sites in Pyeno and ubiquitin (PY03971) respectively, suggests that ubiquitination may be dependent upon prior phosphorylation in response to some extracellular or developmental stimuli [60]. Thus, phosphorylation could be a recognition requirement of the ubiquitin ligating enzyme E3 as has been reported in other organisms [61,62]. Like many other PTMs, ubiquitination of proteins may also be tightly regulated. T7 phosphorylation of PY03971 is the first PTM to be reported in this protein that may be relevant for ubiquitination of neighboring residue K6.

### Conclusion

Analysis of food vacuole proteome from *P* *. yoelii* and its comparison with yeast vacuolar proteome led to identification of several putative orthologs indicating the underlying similarity between the vesicular pathways in the two organisms. Structure of high molecular weight variants of food vacuole associated *P* *. yoelii* enolase was determined using immuno-precipitation along with western analysis and MS/MS experiments. Results showed the conjugation of a tri-ubiquitin moiety to K147 of Pyeno. In all, six different PTMs were identified in *Plasmodium* enolase. Ubiquitination of enolase and phosphorylation of *Plasmodium* *yoelii* ubiquitin moiety (PY03971) at T7 have been reported for the first time. Co-localization of MSP1 and Pfeno on merozoite surface and the similarity in the localization of MSP1, HDP and enolase on hemozoin in food vacuole are described. It is suggested that the low molecular mass form (~50 kDa) of Pyeno may be involved in vacuolar fusion (with analogy from yeast) while triubiquitinated form (75 kDa) may be involved in hemozoin related functions.

## Supporting Information

Figure S1 **MS/MS spectrum of a peptide containing acK384 from *P* *. yoelii* enolase (Pyeno).**
Peptide sequence is ^-366^VNQIGSITEAIEACLLSQKNNWGVMVSHR^394-^.
(PPTX)

Figure S2 **MS/MS spectra of peptides present in ~65-80 kDa gel band digest that have originated from ubiquitinated Pyeno.**
Peptides belong to PY03971 derived ubiquitin moieties. (A) -^12^ TITLDVEPSDTIENVK^27^- (marked in ‘blue’ in Figure 3A); (B) -^55^ TLSDYNIQK^63^- (marked in ‘red’ in Figure 3A); (C)-^64^ESTLHLVLR^72^-(marked in ‘magenta’ in Figure 3A).
(PPTX)

Figure S3 **MS/MS spectra of peptides containing S42 from *P. falciparum* enolase (Pfeno).**
Peptide sequence is -^35^AAVPSGAS42TGIYEALELRDNDKSR^58^-. (A) phosphorylated at S42 (pS) and (B) un-modified peptide.
(PPTX)

Figure S4 **MS/MS spectra of peptides containing T337 from *P* *. yoelii* enolase (Pyeno).**
Peptide sequence is -^323^DVQIVGDDLLVTNPTR^338^-. (A) phosphorylated at T337 (pT) and (B) un-modified peptide.
(PPTX)

Figure S5 **3D structures of Pyeno and Pfeno were modeled on the basis of X-ray structure of *T. gondii* enolase (pdb: 3OTR).**
Residues that are post translationally modified are marked in stick and ball representation (ac-acetylation; u- ubiquitination; p- phosphorylation).
(PPTX)

Figure S6 **Co-localization of MSP1 and enolase on *P. falciparum* in a mature schizont (meozoites) cell surface.**
Polyclonal anti r-Pfeno antibody raised in mouse was used for Pfeno (green) and 1G3 monoclonal antibody (red) that recognizes the MSP1_33_. (a) DAPI; (b) DAPI + MSP1; (c) DAPI + Pfeno; (d) Pfeno + MSP1.
(PPTX)

Figure S7 **Comparison of PTMs in human ENO1 and Pyeno.**
Note that the residues modified in parasite enolase are note conserved in human enzyme (except active site S). This offers an opportunity for selective targeting of *Plasmodium* enolase.
(PPTX)

Table S1 **List of proteins identified in food vacuole preparation made from *P* *. yoelii* as described in Materials & Methods.**
In all 298 proteins could be identified. Many of these proteins represent trace contaminations from other parasite organelles.
(DOCX)

Table S2 **Candidate food vacuole associated proteins.**
Proteins that are likely to be vacuole associated ones (by analogy with yeast) are listed in three classes: (i) includes vacuole resident proteins (e.g. FV proteases and membrane transporters); (ii) involved in vesicular trafficking and (iii) includes proteasomal proteins, homologs of which have been reported to be associated with yeast vacuoles [34]..
(DOCX)
