# Supplementary figures and images for: Food Vacuole Associated Enolase in Plasmodium Undergoes Multiple Post-Translational Modifications: Evidence for Atypical Ubiquitination
Source: PLoS One. 2013 Aug 23;8(8):e72687. doi: 10.1371/journal.pone.0072687 (PMC3751847; doi:10.1371/journal.pone.0072687)

## Slide 1
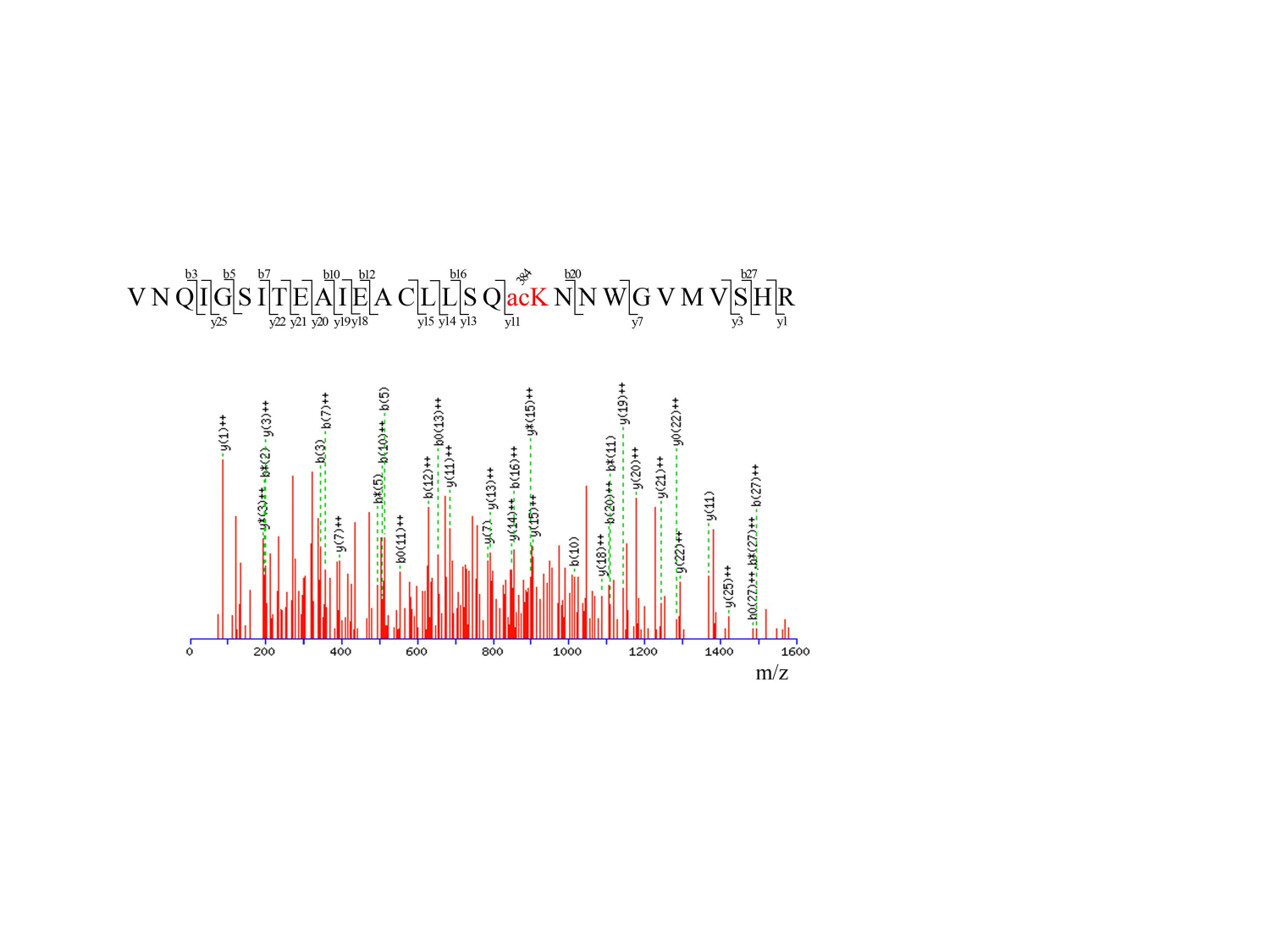

Supplement: Figure S1 — MS/MS spectrum of a peptide containing acK384 from P . yoelii enolase (Pyeno). Peptide sequence is -366VNQIGSITEAIEACLLSQKNNWGVMVSHR394-. (PPTX) [file pone.0072687.s001.pptx]

## Slide 1
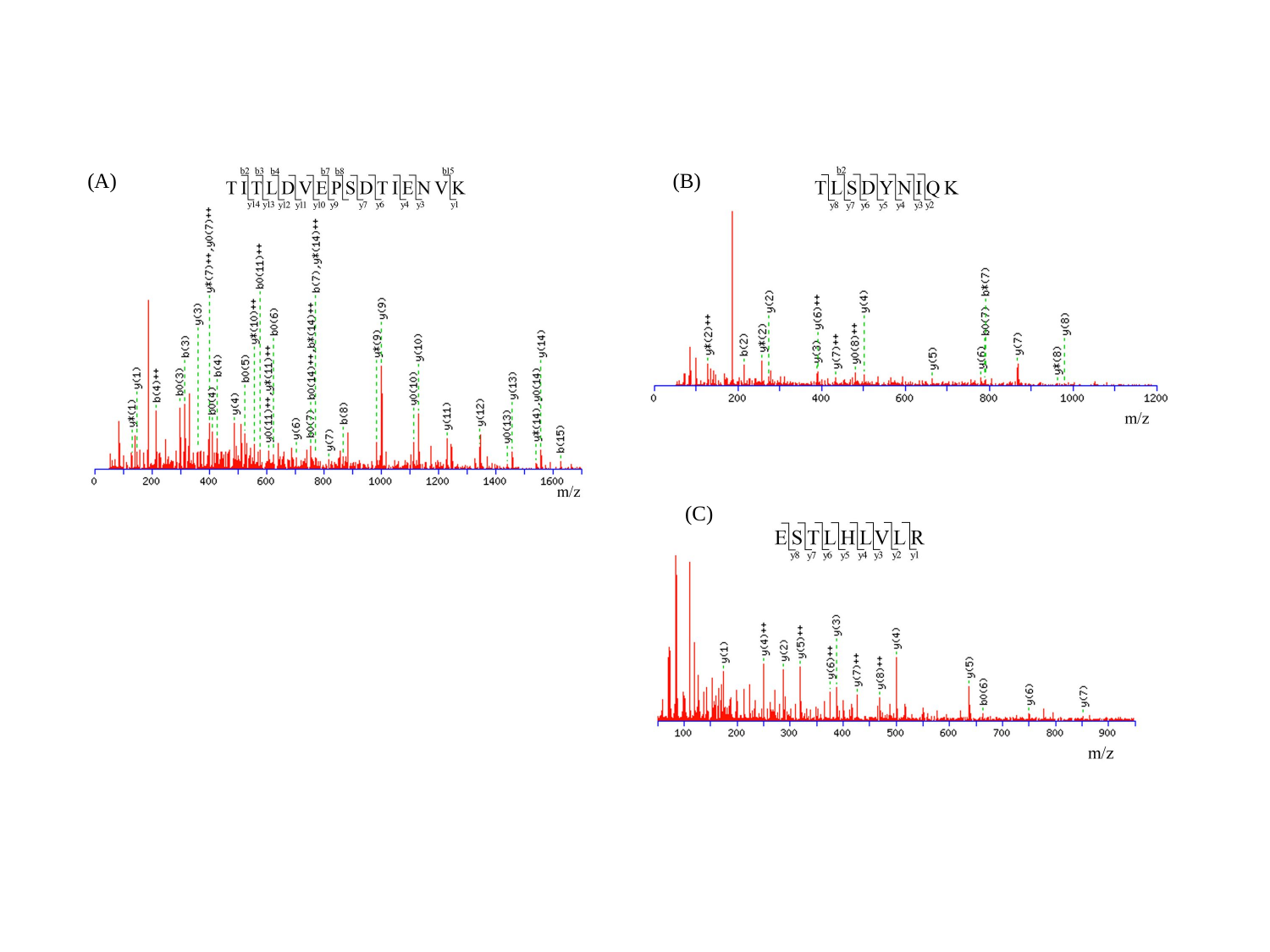

(A)
(B)
(C)

Supplement: Figure S2 — MS/MS spectra of peptides present in ~65-80 kDa gel band digest that have originated from ubiquitinated Pyeno. Peptides belong to PY03971 derived ubiquitin moieties. (A) -12 TITLDVEPSDTIENVK27- (marked in ‘blue’ in Figure 3A); (B) -55 TLSDYNIQK63- (marked in ‘red’ in Figure 3A); (C)-64ESTLHLVLR72-(marked in ‘magenta’ in Figure 3A). (PPTX) [file pone.0072687.s002.pptx]

## Slide 1
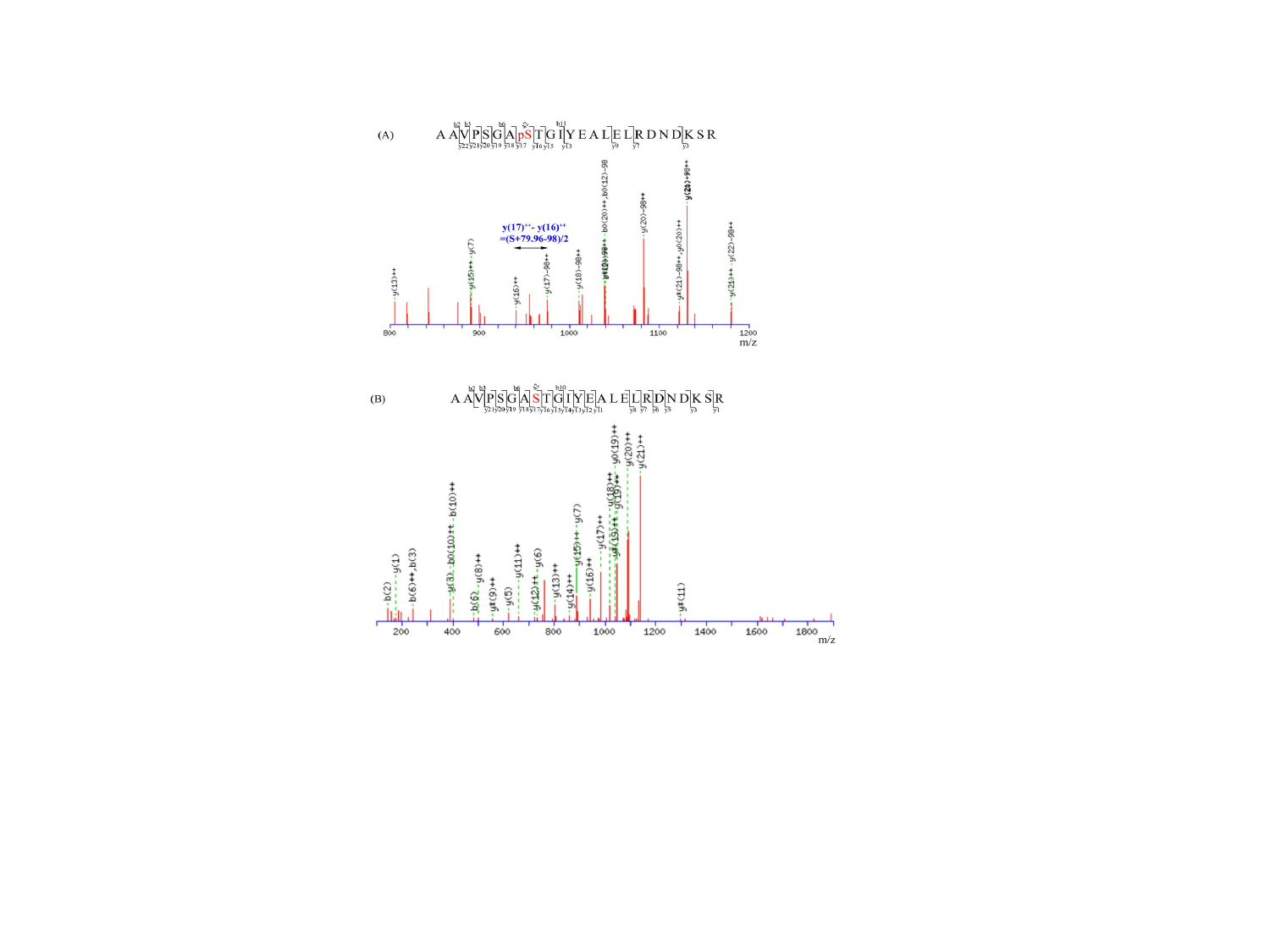

Supplement: Figure S3 — MS/MS spectra of peptides containing S42 from P. falciparum enolase (Pfeno). Peptide sequence is -35AAVPSGAS42TGIYEALELRDNDKSR58-. (A) phosphorylated at S42 (pS) and (B) un-modified peptide. (PPTX) [file pone.0072687.s003.pptx]

## Slide 1
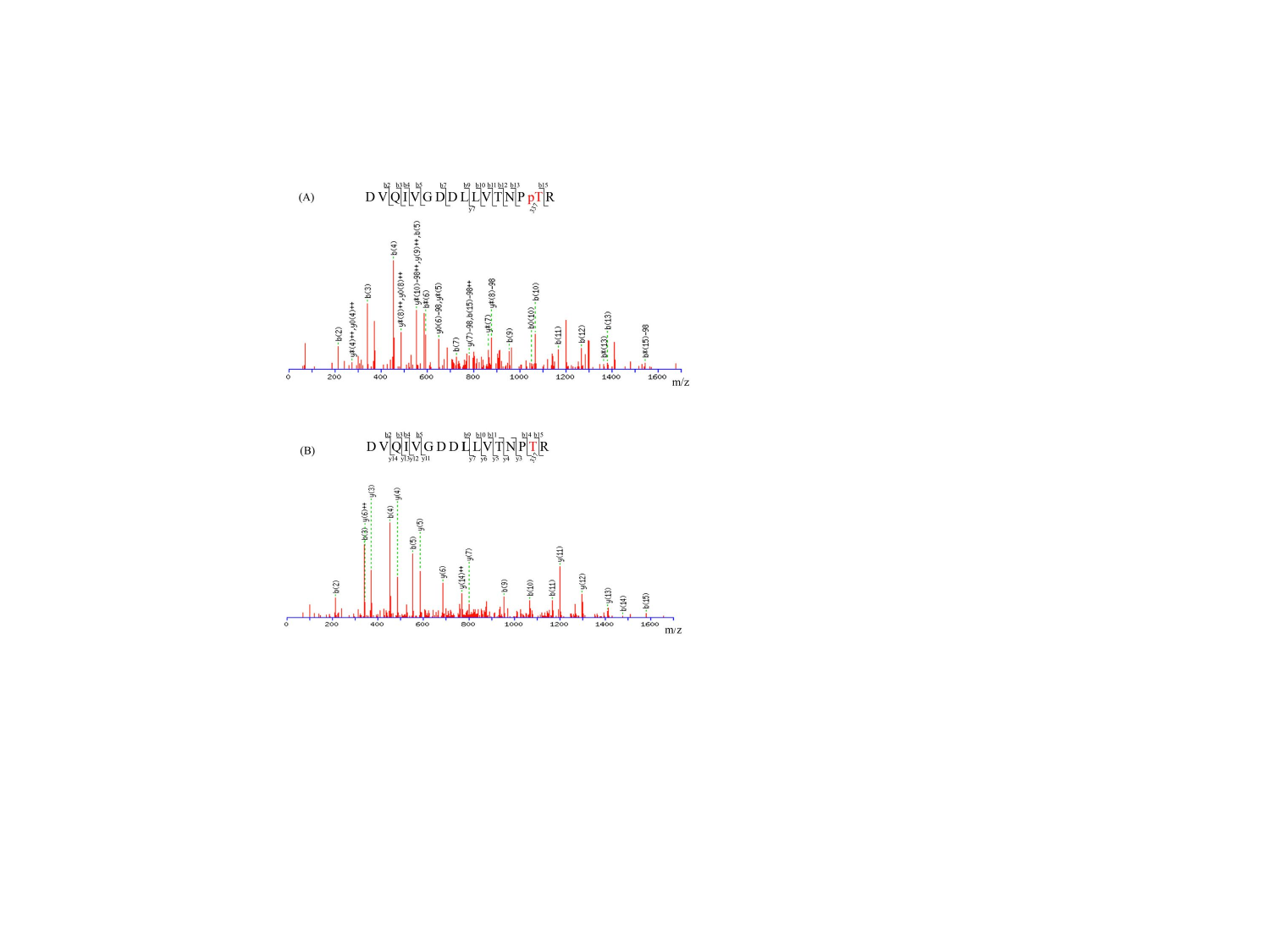

Supplement: Figure S4 — MS/MS spectra of peptides containing T337 from P . yoelii enolase (Pyeno). Peptide sequence is -323DVQIVGDDLLVTNPTR338-. (A) phosphorylated at T337 (pT) and (B) un-modified peptide. (PPTX) [file pone.0072687.s004.pptx]

## Slide 1
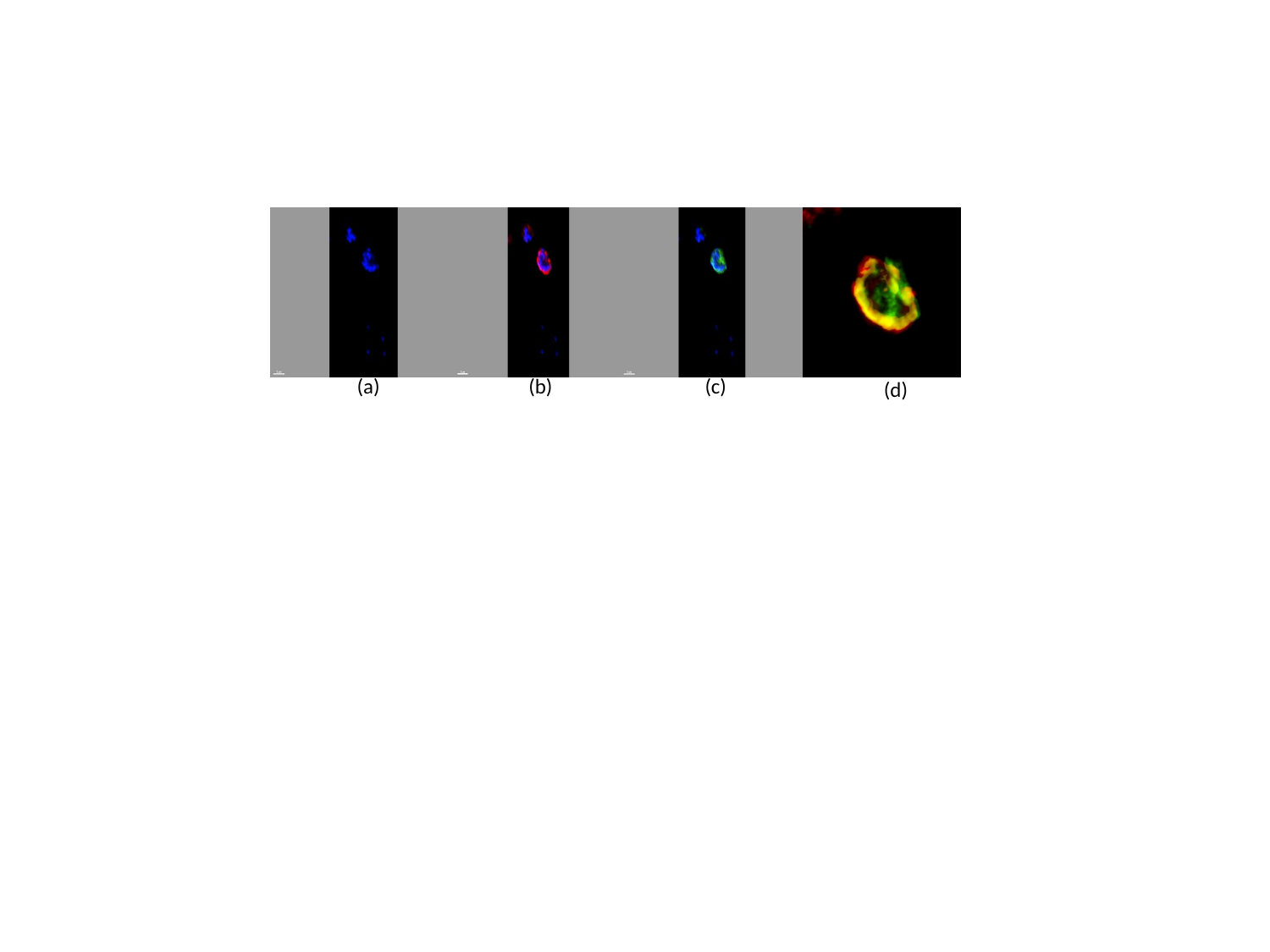

(a)
(b)
(c)
(d)

Supplement: Figure S6 — Co-localization of MSP1 and enolase on P. falciparum in a mature schizont (meozoites) cell surface. Polyclonal anti r-Pfeno antibody raised in mouse was used for Pfeno (green) and 1G3 monoclonal antibody (red) that recognizes the MSP133. (a) DAPI; (b) DAPI + MSP1; (c) DAPI + Pfeno; (d) Pfeno + MSP1. (PPTX) [file pone.0072687.s006.pptx]
