# Supplementary material for: Food Vacuole Associated Enolase in Plasmodium Undergoes Multiple Post-Translational Modifications: Evidence for Atypical Ubiquitination
Source: PLoS One. 2013 Aug 23;8(8):e72687. doi: 10.1371/journal.pone.0072687 (PMC3751847; doi:10.1371/journal.pone.0072687)
Supplement: Figure S5 — 3D structures of Pyeno and Pfeno were modeled on the basis of X-ray structure of T. gondii enolase (pdb: 3OTR). Residues that are post translationally modified are marked in stick and ball representation (ac-acetylation; u- ubiquitination; p- phosphorylation). (PPTX) [file pone.0072687.s005.pptx]

## Slide 1
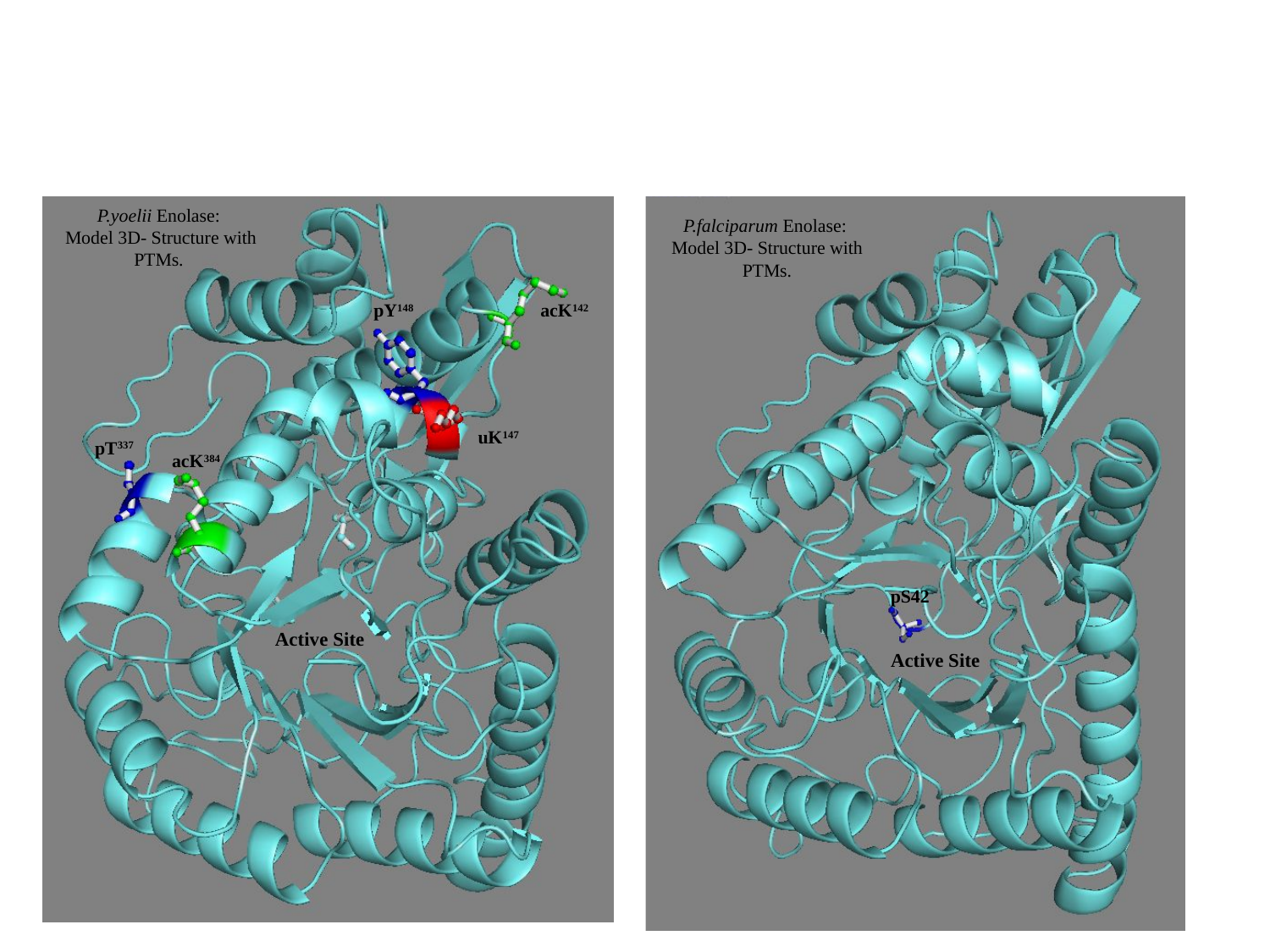

pY148
acK142
uK147
pT337
acK384
Active Site
P.yoelii Enolase:
 Model 3D- Structure with PTMs.
P.falciparum Enolase:
Model 3D- Structure with PTMs.
pS42
Active Site
