# Supplementary material for: Food Vacuole Associated Enolase in Plasmodium Undergoes Multiple Post-Translational Modifications: Evidence for Atypical Ubiquitination
Source: PLoS One. 2013 Aug 23;8(8):e72687. doi: 10.1371/journal.pone.0072687 (PMC3751847; doi:10.1371/journal.pone.0072687)
Supplement: Figure S7 — Comparison of PTMs in human ENO1 and Pyeno. Note that the residues modified in parasite enolase are note conserved in human enzyme (except active site S). This offers an opportunity for selective targeting of Plasmodium enolase. (PPTX) [file pone.0072687.s007.pptx]

## Slide 1
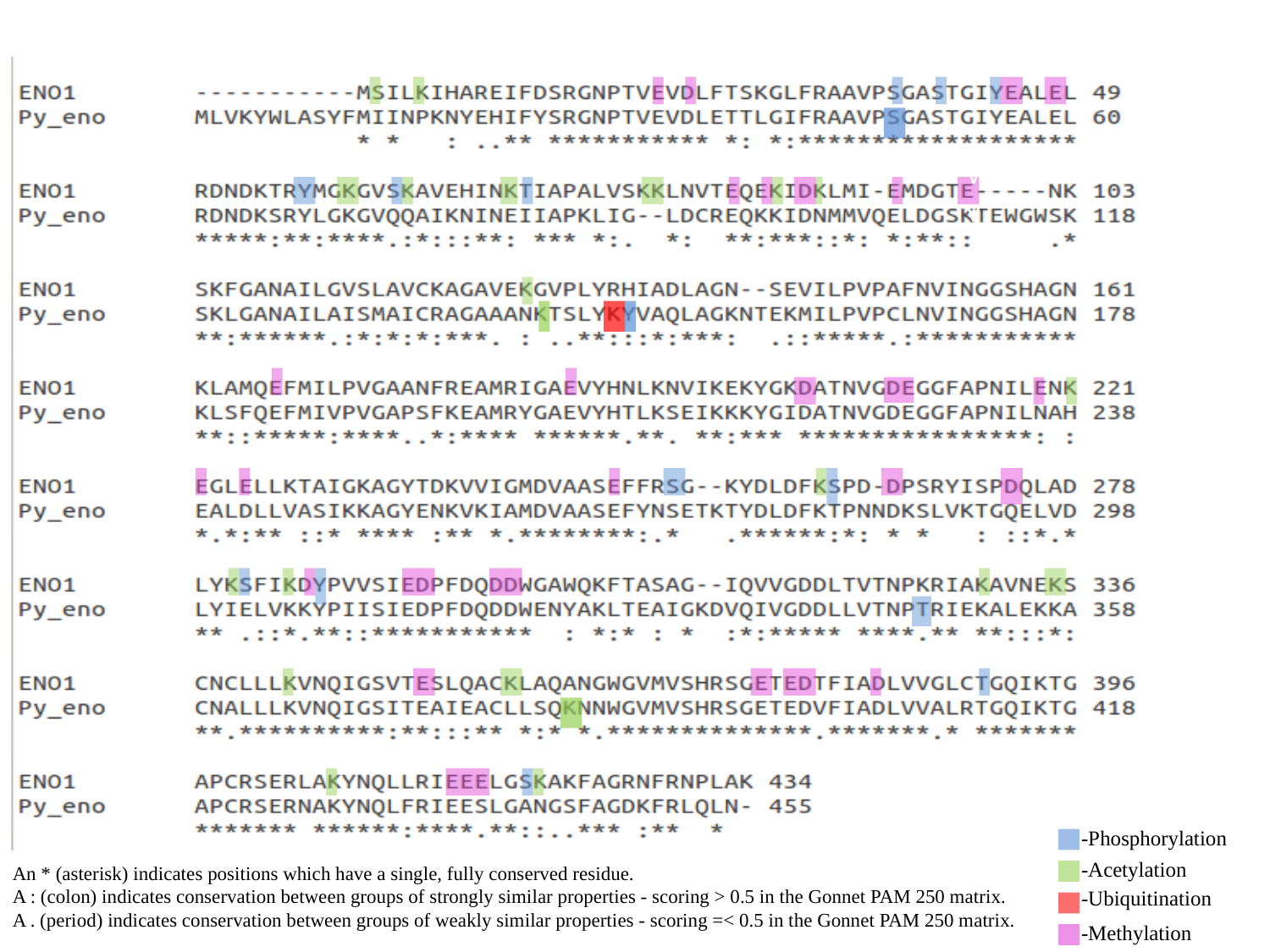

vv
-Phosphorylation
-Acetylation
-Ubiquitination
-Methylation
An * (asterisk) indicates positions which have a single, fully conserved residue. 
A : (colon) indicates conservation between groups of strongly similar properties - scoring > 0.5 in the Gonnet PAM 250 matrix. 
A . (period) indicates conservation between groups of weakly similar properties - scoring =< 0.5 in the Gonnet PAM 250 matrix.
