# Supplementary material for: Food Vacuole Associated Enolase in Plasmodium Undergoes Multiple Post-Translational Modifications: Evidence for Atypical Ubiquitination
Source: PLoS One. 2013 Aug 23;8(8):e72687. doi: 10.1371/journal.pone.0072687 (PMC3751847; doi:10.1371/journal.pone.0072687)
Supplement: Table S1 — List of proteins identified in food vacuole preparation made from P . yoelii as described in Materials & Methods. In all 298 proteins could be identified. Many of these proteins represent trace contaminations from other parasite organelles. (DOCX) [file pone.0072687.s008.docx]

**Table S1: List of proteins identified in food vacuole preparation made from *P. yoelii* as described in Methods & Materials.** ________________________________________________________________________

| **Sr. No.** | **NCBI protein accession** | **Protein Description** | **Protein score** | **Protein cover** |
| --- | --- | --- | --- | --- |
| 1 | gi\|83317521 | 19 kDa sporozoite antigen [Plasmodium yoelii yoelii str. 17XNL] | 127 | 29.4 |
| 2 | gi\|68531997 | 2-oxoglutarate dehydrogenase, E2 component, dihydrolipoamide succinyltransferase [Plasmodium yoelii yoelii str. 17XNL] | 20 | 4.2 |
| 3 | gi\|82596054 | 26s protease regulatory subunit 6a [Plasmodium yoelii yoelii str. 17XNL] | 65 | 24.7 |
| 4 | gi\|83314741 | 26s protease regulatory subunit s10b [Plasmodium yoelii yoelii str. 17XNL] | 45 | 22.6 |
| 5 | gi\|82593910 | 26S proteasome ATPase [Plasmodium yoelii yoelii str. 17XNL] | 85 | 13.5 |
| 6 | gi\|82594499 | 26s proteasome regulatory subunit s12 [Plasmodium yoelii yoelii str. 17XNL] | 55 | 5.3 |
| 7 | gi\|82915518 | 26S proteasome subunit [Plasmodium yoelii yoelii str. 17XNL] | 55 | 37.6 |
| 8 | gi\|82704989 | 26S proteasome subunit 4 protein [Plasmodium yoelii yoelii str. 17XNL] | 31 | 15.4 |
| 9 | gi\|82541772 | 26S proteasome subunit P40.5 [Plasmodium yoelii yoelii str. 17XNL] | 120 | 53.3 |
| 10 | gi\|83315300 | 26S proteasome subunit P45 [Plasmodium yoelii yoelii str. 17XNL] | 25 | 7.8 |
| 11 | gi\|82596443 | 40S ribosomal protein S12 [Plasmodium yoelii yoelii str. 17XNL] | 25 | 7.7 |
| 12 | gi\|83273502 | 40s ribosomal protein s14 [Plasmodium yoelii yoelii str. 17XNL] | 43 | 31.4 |
| 13 | gi\|83286443 | 40S ribosomal protein S3a [Plasmodium yoelii yoelii str. 17XNL] | 46 | 24.3 |
| 14 | gi\|83315577 | 60S acidic ribosomal protein P2 [Plasmodium yoelii yoelii str. 17XNL] | 199 | 67.6 |
| 15 | gi\|68075317 | 60S ribosomal protein L11a [Plasmodium berghei strain ANKA] | 38 | 27.2 |
| 16 | gi\|83286156 | 60S ribosomal protein L14 [Plasmodium yoelii yoelii str. 17XNL] | 78 | 30.5 |
| 17 | gi\|82541351 | 60S ribosomal protein L23a [Plasmodium yoelii yoelii str. 17XNL] | 31 | 18.1 |
| 18 | gi\|83273786 | 60S ribosomal protein L27 [Plasmodium yoelii yoelii str. 17XNL] | 49 | 6.6 |
| 19 | gi\|82594928 | 60S ribosomal protein L30 [Plasmodium yoelii yoelii str. 17XNL] | 57 | 26.9 |
| 20 | gi\|83315079 | 60S ribosomal protein L6 [Plasmodium yoelii yoelii str. 17XNL] | 128 | 49.1 |
| 21 | gi\|82596445 | 60S ribosomal protein L7 [Plasmodium yoelii yoelii str. 17XNL] | 52 | 10.9 |
| 22 | gi\|82705796 | 60S ribosomal protein L7a [Plasmodium yoelii yoelii str. 17XNL] | 51 | 23.9 |
| 23 | gi\|82540649 | 60S ribosomal protein L8 [Plasmodium yoelii yoelii str. 17XNL] | 58 | 15.8 |
| 24 | gi\|83286320 | actin [Plasmodium yoelii yoelii str. 17XNL] | 72 | 39.4 |
| 25 | gi\|82541602 | actin depolymerizing factor [Plasmodium yoelii yoelii str. 17XNL] | 40 | 18 |
| 26 | gi\|82539417 | adenine nucleotide translocase [Plasmodium yoelii yoelii str. 17XNL] | 86 | 31.2 |
| 27 | gi\|83285917 | adenosine deaminase [Plasmodium yoelii yoelii str. 17XNL] | 223 | 66.5 |
| 28 | gi\|83315551 | adenosylhomocysteinase [Plasmodium yoelii yoelii str. 17XNL] | 65 | 12.1 |
| 29 | gi\|82541272 | ADP-ribosylation factor [Plasmodium yoelii yoelii str. 17XNL] | 65 | 49.7 |
| 30 | gi\|82539264 | ADP-ribosylation factor GTPase-activating protein [Plasmodium yoelii yoelii str. 17XNL] | 32 | 24.3 |
| 31 | gi\|3676478 | ADP-ribosylation factor GTPase-activating protein [Plasmodium yoelii] | 32 | 24.3 |
| 32 | gi\|83033179 | apical membrane antigen-1 [Plasmodium yoelii yoelii str. 17XNL] | 30 | 12.4 |
| 33 | gi\|151933941 | apical membrane antigen-1 [Plasmodium yoelii yoelii] | 30 | 33.8 |
| 34 | gi\|83314502 | asparaginyl-tRNA synthetase [Plasmodium yoelii yoelii str. 17XNL] | 42 | 5.3 |
| 35 | gi\|82705063 | aspartate carbamoyltransferase [Plasmodium yoelii yoelii str. 17XNL] | 141 | 38.6 |
| 36 | gi\|82597098 | ATP synthase F1 subunit alpha [Plasmodium yoelii yoelii str. 17XNL] | 44 | 15.3 |
| 37 | gi\|83316096 | ATP synthase F1 subunit delta [Plasmodium yoelii yoelii str. 17XNL] | 56 | 14.5 |
| 38 | gi\|83315296 | ATP synthase F1 subunit gamma [Plasmodium yoelii yoelii str. 17XNL] | 30 | 10.4 |
| 39 | gi\|82793401 | ATP synthase F1subunit beta [Plasmodium yoelii yoelii str. 17XNL] | 35 | 16.9 |
| 40 | gi\|82540811 | ATP synthase subunit [Plasmodium yoelii yoelii str. 17XNL] | 35 | 11.5 |
| 41 | gi\|82596078 | ATPase, AAA family [Plasmodium yoelii yoelii str. 17XNL] | 26 | 3.2 |
| 42 | gi\|82538952 | bisphosphate aldolase class-I [Plasmodium yoelii yoelii str. 17XNL] | 37 | 23 |
| 43 | gi\|82594584 | branched-chain alpha-keto acid dehydrogenase E1-alpha subunit [Plasmodium yoelii yoelii str. 17XNL] | 30 | 41.3 |
| 44 | gi\|82753752 | caltractin [Plasmodium yoelii yoelii str. 17XNL] | 25 | 9.5 |
| 45 | gi\|82704503 | casein kinase i [Plasmodium yoelii yoelii str. 17XNL] | 58 | 22.6 |
| 46 | gi\|82794069 | CCAAT-box DNA binding protein subunit B [Plasmodium yoelii yoelii str. 17XNL] | 24 | 3.7 |
| 47 | gi\|82793784 | CCT chaperonin subunit gamma [Plasmodium yoelii yoelii str. 17XNL] | 61 | 27.1 |
| 48 | gi\|82538797 | cell division cycle protein 48 [Plasmodium yoelii yoelii str. 17XNL] | 288 | 55.5 |
| 49 | gi\|70945830 | centrin [Plasmodium chabaudi chabaudi] | 25 | 11.4 |
| 50 | gi\|78101383 | Chain A, Crystal Structure Of Plasmodium Yoelii Adenosine Deaminase (Py02076) | 223 | 64.4 |
| 51 | gi\|83286360 | chaperonin [Plasmodium yoelii yoelii str. 17XNL] | 67 | 20.9 |
| 52 | gi\|83315670 | chaperonin containing TCP-1 subunit delta [Plasmodium yoelii yoelii str. 17XNL] | 61 | 22.2 |
| 53 | gi\|82541751 | chaperonin Cpn60 [Plasmodium yoelii yoelii str. 17XNL] | 58 | 40.5 |
| 54 | gi\|83282437 | chaperonin, 60 kDa [Plasmodium yoelii yoelii str. 17XNL] | 36 | 5.9 |
| 55 | gi\|83282359 | chromosome condensation regulator leucine aminopeptidase [Plasmodium yoelii yoelii str. 17XNL] | 64 | 11.2 |
| 56 | gi\|82705747 | co-chaperone GrpE [Plasmodium yoelii yoelii str. 17XNL] | 37 | 5.3 |
| 57 | gi\|83317352 | Cof-like hydrolase [Plasmodium yoelii yoelii str. 17XNL] | 84 | 31.1 |
| 58 | gi\|83286741 | conjugation stage-specific protein [Plasmodium yoelii yoelii str. 17XNL] | 29 | 20.3 |
| 59 | gi\|83315601 | cysteine protease [Plasmodium yoelii yoelii str. 17XNL] | 69 | 1.1 |
| 60 | gi\|83317695 | cytochrome c1, heme protein [Plasmodium yoelii yoelii str. 17XNL] | 21 | 4.6 |
| 61 | gi\|83317473 | DEAD/DEAH box helicase [Plasmodium yoelii yoelii str. 17XNL] | 55 | 14.3 |
| 62 | gi\|82539543 | developmentally regulated GTP-binding protein 2 [Plasmodium yoelii yoelii str. 17XNL] | 20 | 4.6 |
| 63 | gi\|15429020 | drug resistance protein MDR1 [Plasmodium yoelii] | 26 | 9.1 |
| 64 | gi\|82913288 | dynamin protein [Plasmodium yoelii yoelii str. 17XNL] | 75 | 3.7 |
| 65 | gi\|17981645 | dynamin-like protein [Plasmodium yoelii yoelii] | 75 | 1.8 |
| 66 | gi\|82754026 | elongation factor 1-gamma 1 [Plasmodium yoelii yoelii str. 17XNL] | 72 | 27.9 |
| 67 | gi\|82752500 | enolase [Plasmodium yoelii yoelii str. 17XNL] | 334 | 69.7 |
| 68 | gi\|82705642 | ER lumen protein retaining receptor protein [Plasmodium yoelii yoelii str. 17XNL] | 29 | 5.3 |
| 69 | gi\|82595599 | erythrocyte membrane protein [Plasmodium yoelii yoelii str. 17XNL] | 18 | 0.7 |
| 70 | gi\|82541004 | ethylene-inducible protein hever [Plasmodium yoelii yoelii str. 17XNL] | 50 | 18.2 |
| 71 | gi\|82594992 | eukaryotic DNA topoisomerase I [Plasmodium yoelii yoelii str. 17XNL] | 27 | 0.8 |
| 72 | gi\|83032673 | eukaryotic ribosomal protein L18 [Plasmodium yoelii yoelii str. 17XNL] | 95 | 20.6 |
| 73 | gi\|82793922 | eukaryotic translation initiation factor 2 subunit alpha [Plasmodium yoelii yoelii str. 17XNL] | 39 | 8.9 |
| 74 | gi\|83285950 | eukaryotic translation initiation factor 2, subunit 2 [Plasmodium yoelii yoelii str. 17XNL] | 29 | 14.7 |
| 75 | gi\|82594751 | eukaryotic translation initiation factor 3 39 kDa subunit [Plasmodium yoelii yoelii str. 17XNL] | 58 | 34.6 |
| 76 | gi\|82593902 | eukaryotic translation initiation factor 3 p42 [Plasmodium yoelii yoelii str. 17XNL] | 65 | 9.3 |
| 77 | gi\|83282221 | eukaryotic translation initiation factor 6 protein [Plasmodium yoelii yoelii str. 17XNL] | 41 | 6.9 |
| 78 | gi\|82595178 | FAD-dependent glycerol-3-phosphate dehydrogenase [Plasmodium yoelii yoelii str. 17XNL] | 157 | 54.2 |
| 79 | gi\|13877307 | fructose 1,6-bisphosphate aldolase [Plasmodium yoelii] | 37 | 21.9 |
| 80 | gi\|82541573 | glutamine synthetase [Plasmodium yoelii yoelii str. 17XNL] | 39 | 4.3 |
| 81 | gi\|83286175 | glutaredoxin protein [Plasmodium yoelii yoelii str. 17XNL] | 27 | 13 |
| 82 | gi\|83317699 | glyceraldehyde-3-phosphate dehydrogenase [Plasmodium yoelii yoelii str. 17XNL] | 291 | 47.2 |
| 83 | gi\|82540395 | GTP-binding nuclear protein RAN/TC4 [Plasmodium yoelii yoelii str. 17XNL] | 89 | 58.8 |
| 84 | gi\|82540698 | guanylyl cyclase [Plasmodium yoelii yoelii str. 17XNL] | 39 | 2.3 |
| 85 | gi\|83032705 | heat shock 90 kDa protein [Plasmodium yoelii yoelii str. 17XNL] | 57 | 47.1 |
| 86 | gi\|82594363 | heat shock protein [Plasmodium yoelii yoelii str. 17XNL] | 283 | 56.8 |
| 87 | gi\|82541395 | heat shock protein 60 [Plasmodium yoelii yoelii str. 17XNL] | 209 | 38.3 |
| 88 | gi\|3885993 | heat shock protein 60 [Plasmodium yoelii] | 209 | 36.8 |
| 89 | gi\|82704924 | heat shock protein 70 [Plasmodium yoelii yoelii str. 17XNL] | 382 | 41.5 |
| 90 | gi\|83032707 | heat shock protein 81-2 [Plasmodium yoelii yoelii str. 17XNL] | 201 | 53.1 |
| 91 | gi\|83282377 | heat shock protein 83 [Plasmodium yoelii yoelii str. 17XNL] | 78 | 10.1 |
| 92 | gi\|82594539 | heat shock protein 90 [Plasmodium yoelii yoelii str. 17XNL] | 262 | 38.4 |
| 93 | gi\|83282486 | heat shock protein ATPase subunit HslU [Plasmodium yoelii yoelii str. 17XNL] | 25 | 4.2 |
| 94 | gi\|82793288 | heat shock protein DnaJ [Plasmodium yoelii yoelii str. 17XNL] | 41 | 3.8 |
| 95 | gi\|82753607 | heat shock protein hsp70 [Plasmodium yoelii yoelii str. 17XNL] | 190 | 48.6 |
| 96 | gi\|82705409 | high molecular weight rhoptry protein 3 [Plasmodium yoelii yoelii str. 17XNL] | 89 | 23.8 |
| 97 | gi\|11862911 | high molecular weight rhoptry protein 3 [Plasmodium yoelii] | 332 | 58 |
| 98 | gi\|82541094 | histone 3 [Plasmodium yoelii yoelii str. 17XNL] | 29 | 19.1 |
| 99 | gi\|82594560 | histone H2a [Plasmodium yoelii yoelii str. 17XNL] | 76 | 35.3 |
| 100 | gi\|82539816 | histone H2A variant [Plasmodium yoelii yoelii str. 17XNL] | 79 | 38.3 |
| 101 | gi\|83273818 | histone-binding protein n1/n2 [Plasmodium yoelii yoelii str. 17XNL] | 28 | 10.6 |
| 102 | gi\|68531968 | hypothetical protein [Plasmodium yoelii yoelii str. 17XNL] | 34 | 5.7 |
| 103 | gi\|68532002 | hypothetical protein [Plasmodium yoelii yoelii str. 17XNL] | 39 | 3.9 |
| 104 | gi\|68532068 | hypothetical protein [Plasmodium yoelii yoelii str. 17XNL] | 43 | 12.4 |
| 105 | gi\|68532072 | hypothetical protein [Plasmodium yoelii yoelii str. 17XNL] | 32 | 3.4 |
| 106 | gi\|81177644 | hypothetical protein [Plasmodium yoelii yoelii str. 17XNL] | 25 | 26.7 |
| 107 | gi\|82538896 | hypothetical protein [Plasmodium yoelii yoelii str. 17XNL] | 65 | 21.5 |
| 108 | gi\|82539279 | hypothetical protein [Plasmodium yoelii yoelii str. 17XNL] | 38 | 18 |
| 109 | gi\|82539307 | hypothetical protein [Plasmodium yoelii yoelii str. 17XNL] | 36 | 13.5 |
| 110 | gi\|82539396 | hypothetical protein [Plasmodium yoelii yoelii str. 17XNL] | 40 | 5.5 |
| 111 | gi\|82539519 | hypothetical protein [Plasmodium yoelii yoelii str. 17XNL] | 77 | 23.1 |
| 112 | gi\|82539669 | hypothetical protein [Plasmodium yoelii yoelii str. 17XNL] | 40 | 12.2 |
| 113 | gi\|82539837 | hypothetical protein [Plasmodium yoelii yoelii str. 17XNL] | 50 | 18.5 |
| 114 | gi\|82539909 | hypothetical protein [Plasmodium yoelii yoelii str. 17XNL] | 71 | 34.8 |
| 115 | gi\|82539920 | hypothetical protein [Plasmodium yoelii yoelii str. 17XNL] | 41 | 11.5 |
| 116 | gi\|82539958 | hypothetical protein [Plasmodium yoelii yoelii str. 17XNL] | 31 | 11.3 |
| 117 | gi\|82540016 | hypothetical protein [Plasmodium yoelii yoelii str. 17XNL] | 25 | 15.6 |
| 118 | gi\|82540445 | hypothetical protein [Plasmodium yoelii yoelii str. 17XNL] | 49 | 21.3 |
| 119 | gi\|82540766 | hypothetical protein [Plasmodium yoelii yoelii str. 17XNL] | 78 | 19.2 |
| 120 | gi\|82540937 | hypothetical protein [Plasmodium yoelii yoelii str. 17XNL] | 45 | 8.4 |
| 121 | gi\|82541050 | hypothetical protein [Plasmodium yoelii yoelii str. 17XNL] | 26 | 4.5 |
| 122 | gi\|82541302 | hypothetical protein [Plasmodium yoelii yoelii str. 17XNL] | 24 | 5.8 |
| 123 | gi\|82541449 | hypothetical protein [Plasmodium yoelii yoelii str. 17XNL] | 82 | 77.8 |
| 124 | gi\|82541516 | hypothetical protein [Plasmodium yoelii yoelii str. 17XNL] | 60 | 15.2 |
| 125 | gi\|82541632 | hypothetical protein [Plasmodium yoelii yoelii str. 17XNL] | 76 | 24.1 |
| 126 | gi\|82594098 | hypothetical protein [Plasmodium yoelii yoelii str. 17XNL] | 45 | 40.9 |
| 127 | gi\|82594305 | hypothetical protein [Plasmodium yoelii yoelii str. 17XNL] | 29 | 6 |
| 128 | gi\|82594361 | hypothetical protein [Plasmodium yoelii yoelii str. 17XNL] | 88 | 35.4 |
| 129 | gi\|82594496 | hypothetical protein [Plasmodium yoelii yoelii str. 17XNL] | 81 | 25.1 |
| 130 | gi\|82594712 | hypothetical protein [Plasmodium yoelii yoelii str. 17XNL] | 29 | 1.9 |
| 131 | gi\|82594772 | hypothetical protein [Plasmodium yoelii yoelii str. 17XNL] | 20 | 88 |
| 132 | gi\|82594961 | hypothetical protein [Plasmodium yoelii yoelii str. 17XNL] | 23 | 5.2 |
| 133 | gi\|82594965 | hypothetical protein [Plasmodium yoelii yoelii str. 17XNL] | 30 | 4.6 |
| 134 | gi\|82595159 | hypothetical protein [Plasmodium yoelii yoelii str. 17XNL] | 99 | 24.3 |
| 135 | gi\|82595161 | hypothetical protein [Plasmodium yoelii yoelii str. 17XNL] | 73 | 42.6 |
| 136 | gi\|82595244 | hypothetical protein [Plasmodium yoelii yoelii str. 17XNL] | 35 | 4.9 |
| 137 | gi\|82595251 | hypothetical protein [Plasmodium yoelii yoelii str. 17XNL] | 46 | 24.7 |
| 138 | gi\|82595274 | hypothetical protein [Plasmodium yoelii yoelii str. 17XNL] | 43 | 10.3 |
| 139 | gi\|82595394 | hypothetical protein [Plasmodium yoelii yoelii str. 17XNL] | 44 | 3.1 |
| 140 | gi\|82595449 | hypothetical protein [Plasmodium yoelii yoelii str. 17XNL] | 51 | 3 |
| 141 | gi\|82595457 | hypothetical protein [Plasmodium yoelii yoelii str. 17XNL] | 27 | 15.6 |
| 142 | gi\|82595460 | hypothetical protein [Plasmodium yoelii yoelii str. 17XNL] | 31 | 2.9 |
| 143 | gi\|82595631 | hypothetical protein [Plasmodium yoelii yoelii str. 17XNL] | 37 | 5 |
| 144 | gi\|82595634 | hypothetical protein [Plasmodium yoelii yoelii str. 17XNL] | 19 | 1.8 |
| 145 | gi\|82596172 | hypothetical protein [Plasmodium yoelii yoelii str. 17XNL] | 51 | 8.4 |
| 146 | gi\|82596841 | hypothetical protein [Plasmodium yoelii yoelii str. 17XNL] | 24 | 7.4 |
| 147 | gi\|82596931 | hypothetical protein [Plasmodium yoelii yoelii str. 17XNL] | 52 | 17 |
| 148 | gi\|82704445 | hypothetical protein [Plasmodium yoelii yoelii str. 17XNL] | 33 | 13.7 |
| 149 | gi\|82704912 | hypothetical protein [Plasmodium yoelii yoelii str. 17XNL] | 31 | 15.3 |
| 150 | gi\|82704967 | hypothetical protein [Plasmodium yoelii yoelii str. 17XNL] | 22 | 6.9 |
| 151 | gi\|82705320 | hypothetical protein [Plasmodium yoelii yoelii str. 17XNL] | 35 | 1.7 |
| 152 | gi\|82705342 | hypothetical protein [Plasmodium yoelii yoelii str. 17XNL] | 68 | 28.4 |
| 153 | gi\|82705616 | hypothetical protein [Plasmodium yoelii yoelii str. 17XNL] | 41 | 23.8 |
| 154 | gi\|82705649 | hypothetical protein [Plasmodium yoelii yoelii str. 17XNL] | 27 | 12.2 |
| 155 | gi\|82752978 | hypothetical protein [Plasmodium yoelii yoelii str. 17XNL] | 73 | 41.4 |
| 156 | gi\|82753400 | hypothetical protein [Plasmodium yoelii yoelii str. 17XNL] | 38 | 21 |
| 157 | gi\|82753418 | hypothetical protein [Plasmodium yoelii yoelii str. 17XNL] | 73 | 45 |
| 158 | gi\|82753449 | hypothetical protein [Plasmodium yoelii yoelii str. 17XNL] | 52 | 19.4 |
| 159 | gi\|82793145 | hypothetical protein [Plasmodium yoelii yoelii str. 17XNL] | 30 | 22 |
| 160 | gi\|82794002 | hypothetical protein [Plasmodium yoelii yoelii str. 17XNL] | 55 | 5.7 |
| 161 | gi\|82794296 | hypothetical protein [Plasmodium yoelii yoelii str. 17XNL] | 85 | 28.9 |
| 162 | gi\|82794324 | hypothetical protein [Plasmodium yoelii yoelii str. 17XNL] | 20 | 34.7 |
| 163 | gi\|82794763 | hypothetical protein [Plasmodium yoelii yoelii str. 17XNL] | 22 | 11.4 |
| 164 | gi\|82913396 | hypothetical protein [Plasmodium yoelii yoelii str. 17XNL] | 18 | 0.3 |
| 165 | gi\|82913457 | hypothetical protein [Plasmodium yoelii yoelii str. 17XNL] | 32 | 8.6 |
| 166 | gi\|82914406 | hypothetical protein [Plasmodium yoelii yoelii str. 17XNL] | 50 | 24.9 |
| 167 | gi\|82914775 | hypothetical protein [Plasmodium yoelii yoelii str. 17XNL] | 177 | 23.2 |
| 168 | gi\|82914859 | hypothetical protein [Plasmodium yoelii yoelii str. 17XNL] | 19 | 6.3 |
| 169 | gi\|82915429 | hypothetical protein [Plasmodium yoelii yoelii str. 17XNL] | 23 | 17.3 |
| 170 | gi\|82915516 | hypothetical protein [Plasmodium yoelii yoelii str. 17XNL] | 55 | 20.1 |
| 171 | gi\|83273419 | hypothetical protein [Plasmodium yoelii yoelii str. 17XNL] | 29 | 3.3 |
| 172 | gi\|83273452 | hypothetical protein [Plasmodium yoelii yoelii str. 17XNL] | 61 | 34.1 |
| 173 | gi\|83273695 | hypothetical protein [Plasmodium yoelii yoelii str. 17XNL] | 34 | 2.1 |
| 174 | gi\|83273788 | hypothetical protein [Plasmodium yoelii yoelii str. 17XNL] | 37 | 29.2 |
| 175 | gi\|83273805 | hypothetical protein [Plasmodium yoelii yoelii str. 17XNL] | 27 | 3 |
| 176 | gi\|83282204 | hypothetical protein [Plasmodium yoelii yoelii str. 17XNL] | 226 | 71 |
| 177 | gi\|83282298 | hypothetical protein [Plasmodium yoelii yoelii str. 17XNL] | 43 | 3.1 |
| 178 | gi\|83282329 | hypothetical protein [Plasmodium yoelii yoelii str. 17XNL] | 30 | 3 |
| 179 | gi\|83286419 | hypothetical protein [Plasmodium yoelii yoelii str. 17XNL] | 32 | 3.9 |
| 180 | gi\|83286478 | hypothetical protein [Plasmodium yoelii yoelii str. 17XNL] | 18 | 16 |
| 181 | gi\|83286706 | hypothetical protein [Plasmodium yoelii yoelii str. 17XNL] | 33 | 5.2 |
| 182 | gi\|83314440 | hypothetical protein [Plasmodium yoelii yoelii str. 17XNL] | 27 | 9.9 |
| 183 | gi\|83314442 | hypothetical protein [Plasmodium yoelii yoelii str. 17XNL] | 49 | 3.2 |
| 184 | gi\|83314487 | hypothetical protein [Plasmodium yoelii yoelii str. 17XNL] | 40 | 7.5 |
| 185 | gi\|83314515 | hypothetical protein [Plasmodium yoelii yoelii str. 17XNL] | 58 | 14.8 |
| 186 | gi\|83314657 | hypothetical protein [Plasmodium yoelii yoelii str. 17XNL] | 58 | 15.9 |
| 187 | gi\|83314707 | hypothetical protein [Plasmodium yoelii yoelii str. 17XNL] | 49 | 8.8 |
| 188 | gi\|83314922 | hypothetical protein [Plasmodium yoelii yoelii str. 17XNL] | 28 | 13.9 |
| 189 | gi\|83315560 | hypothetical protein [Plasmodium yoelii yoelii str. 17XNL] | 39 | 22.4 |
| 190 | gi\|83315586 | hypothetical protein [Plasmodium yoelii yoelii str. 17XNL] | 60 | 7.3 |
| 191 | gi\|83315629 | hypothetical protein [Plasmodium yoelii yoelii str. 17XNL] | 52 | 13 |
| 192 | gi\|83315652 | hypothetical protein [Plasmodium yoelii yoelii str. 17XNL] | 73 | 13.5 |
| 193 | gi\|83316018 | hypothetical protein [Plasmodium yoelii yoelii str. 17XNL] | 34 | 10.1 |
| 194 | gi\|83317303 | hypothetical protein [Plasmodium yoelii yoelii str. 17XNL] | 29 | 20 |
| 195 | gi\|83317930 | hypothetical protein [Plasmodium yoelii yoelii str. 17XNL] | 18 | 8.6 |
| 196 | gi\|68525541 | hypoxanthine phosphoribosyltransferase [Plasmodium yoelii yoelii str. 17XNL] | 194 | 21.9 |
| 197 | gi\|82595793 | ibosomal protein L1p [Plasmodium yoelii yoelii str. 17XNL] | 46 | 26.2 |
| 198 | gi\|83273891 | impotin alpha 1b [Plasmodium yoelii yoelii str. 17XNL] | 37 | 18 |
| 199 | gi\|83314941 | integral membrane protein [Plasmodium yoelii yoelii str. 17XNL] | 54 | 18.9 |
| 200 | gi\|83033150 | karyopherin beta [Plasmodium yoelii yoelii str. 17XNL] | 109 | 15.5 |
| 201 | gi\|82539424 | L-lactate dehydrogenase [Plasmodium yoelii yoelii str. 17XNL] | 88 | 20.3 |
| 202 | gi\|82915420 | lysyl-tRNA synthetase [Plasmodium yoelii yoelii str. 17XNL] | 43 | 18.1 |
| 203 | gi\|83033115 | m1-family aminopeptidase [Plasmodium yoelii yoelii str. 17XNL] | 28 | 6.4 |
| 204 | gi\|160082 | major merozoite surface antigen [Plasmodium yoelii] | 40 | 2.2 |
| 205 | gi\|160679 | merozoite surface antigen PY230 [Plasmodium yoelii] | 64 | 12.8 |
| 206 | gi\|806391 | merozoite surface protein 1 [Plasmodium yoelii] | 31 | 6.5 |
| 207 | gi\|82596427 | merozoite surface protein 1 precursor [Plasmodium yoelii yoelii str. 17XNL] | 210 | 20.1 |
| 208 | gi\|82705673 | merozoite surface protein precursor [Plasmodium yoelii yoelii str. 17XNL] | 42 | 8.6 |
| 209 | gi\|9957877 | merozoite surface protein precursor [Plasmodium yoelii] | 42 | 7.8 |
| 210 | gi\|83315528 | merozoite surface protein-9 precursor [Plasmodium yoelii yoelii str. 17XNL] | 164 | 27.1 |
| 211 | gi\|83315806 | methionyl-tRNA synthetase [Plasmodium yoelii yoelii str. 17XNL] | 21 | 16.9 |
| 212 | gi\|83282139 | mitochondrial processing peptidase beta subunit [Plasmodium yoelii yoelii str. 17XNL] | 17 | 5.8 |
| 213 | gi\|83314632 | mitochondrial processing peptidase subunit alpha homolog [Plasmodium yoelii yoelii str. 17XNL] | 41 | 13.9 |
| 214 | gi\|82541079 | molecular chaperone DnaJ [Plasmodium yoelii yoelii str. 17XNL] | 36 | 10.6 |
| 215 | gi\|124505817 | mRNA cleavage factor-like protein, putative [Plasmodium falciparum 3D7] | 24 | 9.9 |
| 216 | gi\|83286579 | multicatalytic endopeptidase Y13180 [Plasmodium yoelii yoelii str. 17XNL] | 16 | 20.5 |
| 217 | gi\|83314634 | multidrug resistance protein [Plasmodium yoelii yoelii str. 17XNL] | 40 | 5.2 |
| 218 | gi\|82539741 | myo-inositol-1-phosphate synthase [Plasmodium yoelii yoelii str. 17XNL] | 37 | 21.7 |
| 219 | gi\|2766214 | nucleosome assembly protein [Plasmodium berghei] | 27 | 10.5 |
| 220 | gi\|82539667 | P-type ATPase HAD superfamily, subfamily IC [Plasmodium yoelii yoelii str. 17XNL] | 47 | 8.8 |
| 221 | gi\|82541204 | phosphoglycerate kinase [Plasmodium yoelii yoelii str. 17XNL] | 168 | 75.7 |
| 222 | gi\|82593908 | phosphoglycerate mutase [Plasmodium yoelii yoelii str. 17XNL] | 44 | 13.3 |
| 223 | gi\|82753618 | phosphoribosylpyrophosphate synthetase [Plasmodium yoelii yoelii str. 17XNL] | 136 | 35.4 |
| 224 | gi\|82753379 | plasmepsin [Plasmodium yoelii yoelii str. 17XNL] | 47 | 23.5 |
| 225 | gi\|83273803 | proliferating cell nuclear antigen [Plasmodium yoelii yoelii str. 17XNL] | 115 | 32.8 |
| 226 | gi\|83282659 | prolyl-tRNA synthetase [Plasmodium yoelii yoelii str. 17XNL] | 42 | 24.5 |
| 227 | gi\|82540049 | proteasome activator pa28 subunit beta [Plasmodium yoelii yoelii str. 17XNL] | 51 | 10.1 |
| 228 | gi\|83315915 | proteasome subunit alpha type 2 [Plasmodium yoelii yoelii str. 17XNL] | 45 | 33.6 |
| 229 | gi\|82705582 | proteasome subunit beta [Plasmodium yoelii yoelii str. 17XNL] | 49 | 11.3 |
| 230 | gi\|82752562 | proteasome subunit beta type 2 [Plasmodium yoelii yoelii str. 17XNL] | 19 | 17.9 |
| 231 | gi\|82595601 | protein disulfide isomerase [Plasmodium yoelii yoelii str. 17XNL] | 22 | 6.1 |
| 232 | gi\|82541413 | purine nucleoside phosphorylase [Plasmodium yoelii yoelii str. 17XNL] | 108 | 45.9 |
| 233 | gi\|16444938 | PyRhopH1A [Plasmodium yoelii] | 70 | 3.7 |
| 234 | gi\|26453334 | PyRhopH2 [Plasmodium yoelii] | 273 | 39.6 |
| 235 | gi\|82915378 | pyrophosphate-dependent phosphofructokinase [Plasmodium yoelii yoelii str. 17XNL] | 78 | 8.8 |
| 236 | gi\|82541463 | pyruvate kinase [Plasmodium yoelii yoelii str. 17XNL] | 108 | 29.9 |
| 237 | gi\|82595062 | Rab2 GTPase [Plasmodium yoelii yoelii str. 17XNL] | 54 | 32.4 |
| 238 | gi\|50400235 | RecName: Full=Enolase; AltName: Full=2-phospho-D-glycerate hydro-lyase; AltName: Full=2-phosphoglycerate dehydratase | 334 | 71.4 |
| 239 | gi\|82594604 | replication protein A large subunit [Plasmodium yoelii yoelii str. 17XNL] | 49 | 3.7 |
| 240 | gi\|82595287 | rhoptry associated protein 1 [Plasmodium yoelii yoelii str. 17XNL] | 89 | 25.2 |
| 241 | gi\|5929879 | rhoptry complex polypeptide Rhop-3 [Plasmodium yoelii] | 64 | 15.7 |
| 242 | gi\|83273948 | ribonucleoprotein [Plasmodium yoelii yoelii str. 17XNL] | 43 | 4.1 |
| 243 | gi\|68525530 | ribonucleoside-diphosphate reductase large chain [Plasmodium yoelii yoelii str. 17XNL] | 53 | 20 |
| 244 | gi\|83315314 | ribosomal L15 [Plasmodium yoelii yoelii str. 17XNL] | 42 | 4.4 |
| 245 | gi\|83032675 | ribosomal L18ae protein [Plasmodium yoelii yoelii str. 17XNL] | 45 | 30.4 |
| 246 | gi\|82705794 | ribosomal L18p/L5e family [Plasmodium yoelii yoelii str. 17XNL] | 146 | 44.9 |
| 247 | gi\|82793887 | ribosomal protein L13 [Plasmodium yoelii yoelii str. 17XNL] | 130 | 47.5 |
| 248 | gi\|82705807 | ribosomal protein L13e [Plasmodium yoelii yoelii str. 17XNL] | 26 | 19.1 |
| 249 | gi\|82594760 | ribosomal protein L21e [Plasmodium yoelii yoelii str. 17XNL] | 42 | 13 |
| 250 | gi\|82753814 | ribosomal protein L22 [Plasmodium yoelii yoelii str. 17XNL] | 35 | 6.4 |
| 251 | gi\|82753589 | ribosomal protein L24e [Plasmodium yoelii yoelii str. 17XNL] | 54 | 15.7 |
| 252 | gi\|83317937 | ribosomal protein L27a [Plasmodium yoelii yoelii str. 17XNL] | 31 | 10.1 |
| 253 | gi\|82596814 | ribosomal protein L3 [Plasmodium yoelii yoelii str. 17XNL] | 45 | 16.1 |
| 254 | gi\|82753019 | ribosomal protein L36e [Plasmodium yoelii yoelii str. 17XNL] | 36 | 22.1 |
| 255 | gi\|82705099 | ribosomal protein L4/L1 [Plasmodium yoelii yoelii str. 17XNL] | 165 | 38.7 |
| 256 | gi\|83033193 | ribosomal protein L6 [Plasmodium yoelii yoelii str. 17XNL] | 66 | 43.1 |
| 257 | gi\|83273504 | ribosomal protein S11 [Plasmodium yoelii yoelii str. 17XNL] | 54 | 53.8 |
| 258 | gi\|82539545 | ribosomal protein S15 [Plasmodium yoelii yoelii str. 17XNL] | 38 | 11.9 |
| 259 | gi\|83282342 | ribosomal protein S19 [Plasmodium yoelii yoelii str. 17XNL] | 40 | 11.3 |
| 260 | gi\|82539918 | ribosomal protein S19e [Plasmodium yoelii yoelii str. 17XNL] | 73 | 42.5 |
| 261 | gi\|82704653 | ribosomal protein S2 [Plasmodium yoelii yoelii str. 17XNL] | 66 | 9.9 |
| 262 | gi\|82596441 | ribosomal protein S23 [Plasmodium yoelii yoelii str. 17XNL] | 32 | 22.6 |
| 263 | gi\|83286205 | ribosomal protein S4 [Plasmodium yoelii yoelii str. 17XNL] | 47 | 20.6 |
| 264 | gi\|82539137 | ribosomal protein S4 X isoform [Plasmodium yoelii yoelii str. 17XNL] | 42 | 45.5 |
| 265 | gi\|82752715 | ribosomal protein S5 [Plasmodium yoelii yoelii str. 17XNL] | 89 | 30.2 |
| 266 | gi\|82705619 | ribosomal protein S6e [Plasmodium yoelii yoelii str. 17XNL] | 78 | 22.3 |
| 267 | gi\|82704680 | ribosomal protein S7 [Plasmodium yoelii yoelii str. 17XNL] | 26 | 19.1 |
| 268 | gi\|82539474 | ribosomal protein S7e [Plasmodium yoelii yoelii str. 17XNL] | 95 | 44.3 |
| 269 | gi\|83273616 | ribosomal protein S8e [Plasmodium yoelii yoelii str. 17XNL] | 90 | 37 |
| 270 | gi\|82540018 | ribosomal S17 [Plasmodium yoelii yoelii str. 17XNL] | 63 | 6 |
| 271 | gi\|82540694 | RNA helicase-1 [Plasmodium yoelii yoelii str. 17XNL] | 136 | 23.9 |
| 272 | gi\|82793232 | serine/threonine protein phosphatase alpha-3 isoform [Plasmodium yoelii yoelii str. 17XNL] | 30 | 8.9 |
| 273 | gi\|82540711 | small GTP-binding protein [Plasmodium yoelii yoelii str. 17XNL] | 27 | 64.9 |
| 274 | gi\|82793187 | small GTPase Rab11 [Plasmodium yoelii yoelii str. 17XNL] | 53 | 20.3 |
| 275 | gi\|82594286 | spermidine synthase [Plasmodium yoelii yoelii str. 17XNL] | 55 | 23.1 |
| 276 | gi\|82540657 | splicing factor 1 [Plasmodium yoelii yoelii str. 17XNL] | 36 | 14.2 |
| 277 | gi\|82704506 | structure specific recognition protein [Plasmodium yoelii yoelii str. 17XNL] | 167 | 35.7 |
| 278 | gi\|82753291 | sugar transporter [Plasmodium yoelii yoelii str. 17XNL] | 61 | 5.3 |
| 279 | gi\|82539552 | T-complex protein 1 [Plasmodium yoelii yoelii str. 17XNL] | 89 | 13.8 |
| 280 | gi\|82793596 | T-complex protein 1 subunit alpha [Plasmodium yoelii yoelii str. 17XNL] | 43 | 8.2 |
| 281 | gi\|82594819 | T-complex protein 1 subunit epsilon [Plasmodium yoelii yoelii str. 17XNL] | 44 | 17.8 |
| 282 | gi\|82753168 | T-complex protein subunit beta [Plasmodium yoelii yoelii str. 17XNL] | 39 | 20.6 |
| 283 | gi\|82753605 | thioredoxin [Plasmodium yoelii yoelii str. 17XNL] | 44 | 41.3 |
| 284 | gi\|83315146 | thioredoxin peroxidase 2 [Plasmodium yoelii yoelii str. 17XNL] | 33 | 39.1 |
| 285 | gi\|82704929 | translation elongation factor 1 beta [Plasmodium yoelii yoelii str. 17XNL] | 74 | 42.9 |
| 286 | gi\|81177589 | translation elongation factor EF-1, subunit alpha [Plasmodium yoelii yoelii str. 17XNL] | 184 | 63.2 |
| 287 | gi\|82539835 | translation initiation factor eIF-2 [Plasmodium yoelii yoelii str. 17XNL] | 40 | 18.1 |
| 288 | gi\|82705160 | transmembrane protein 17 [Plasmodium yoelii yoelii str. 17XNL] | 24 | 12.6 |
| 289 | gi\|82596321 | tubulin subunit beta [Plasmodium yoelii yoelii str. 17XNL] | 74 | 28.8 |
| 290 | gi\|82540858 | U43539 hepatocyte erythrocyte protein 17 kDa [Plasmodium yoelii yoelii str. 17XNL] | 32 | 10.1 |
| 291 | gi\|82596094 | ubiquinol-cytochrome c reductase, iron-sulfur subunit [Plasmodium yoelii yoelii str. 17XNL] | 54 | 19.3 |
| 292 | gi\|83317838 | ubiquitin [Plasmodium yoelii yoelii str. 17XNL] | 35 | 19.1 |
| 293 | gi\|82539872 | ubiquitin-like protein Dsk2 [Plasmodium yoelii yoelii str. 17XNL] | 23 | 6.4 |
| 294 | gi\|82753560 | UBP1 interacting protein 2a [Plasmodium yoelii yoelii str. 17XNL] | 23 | 11.3 |
| 295 | gi\|83033113 | V-type ATPase subunit A [Plasmodium yoelii yoelii str. 17XNL] | 68 | 12.9 |
| 296 | gi\|82753243 | V-type H(+)-translocating pyrophosphatase [Plasmodium yoelii yoelii str. 17XNL] | 76 | 6.7 |
| 297 | gi\|82595506 | vacuolar proton translocating ATPase 116 kDa subunit a isoform 1 [Plasmodium yoelii yoelii str. 17XNL] | 21 | 7.7 |
| 298 | gi\|156097643 | variable surface protein Vir22/24-like [Plasmodium vivax SaI-1] | 24 | 5 |

In all 298 proteins could be identified. Many of these proteins represent trace contaminations from other parasite organelles.
