# Supplementary material for: Food Vacuole Associated Enolase in Plasmodium Undergoes Multiple Post-Translational Modifications: Evidence for Atypical Ubiquitination
Source: PLoS One. 2013 Aug 23;8(8):e72687. doi: 10.1371/journal.pone.0072687 (PMC3751847; doi:10.1371/journal.pone.0072687)
Supplement: Table S2 — Candidate food vacuole associated proteins. Proteins that are likely to be vacuole associated ones (by analogy with yeast) are listed in three classes: (i) includes vacuole resident proteins (e.g. FV proteases and membrane transporters); (ii) involved in vesicular trafficking and (iii) includes proteasomal proteins, homologs of which have been reported to be associated with yeast vacuoles [34].. (DOCX) [file pone.0072687.s009.docx]

**Table S2: Candidate food vacuole associated proteins.**.

_____________________________________________________________________

| **NCBI acc. number** | **PlasmoDB gene ID** | **PlasmoDB Protein description** | **Localization/ predicted function** |
| --- | --- | --- | --- |
| *Vacuolar proteases and transporters* | | | |
| gi\|82594361 | PY05000 * | Hypothetical protein | Homologue of Acid phosphatase, an FV resident protein |
| gi\|83033115 | PY01557 * | M1-family aminopeptidase | FV resident protein |
| gi\|83314634 | PY00245 * | Multidrug resistance protein | FV resident protein |
| gi\|74879844 | PY05061 * | Putative chloroquine resistance transporter (cg10) | FV resident protein |
| gi\|82753379 | PY06899 * | Plasmepsin | FV resident protein |
| gi\|83033113 | PY01556 * | V-type ATPase subunit A | FV resident protein |
| gi\|82595506 | PY05420 * | Vacuolar proton translocating ATPase 116 kDa subunit a isoform 1 | FV resident protein |
| gi\|82753243 | PY06858 * | V-type H(+)-translocating pyrophosphatase | Predicted FV resident protein |
| gi\|82540811 | PY04403 * | ATP synthase subunit | homologue of vacuolar ATP synthase subunit d [*P. vivax* SaI-1], a predicted FV resident protein |
| *Proteins involved in vesicular trafficking* | | | |
| gi\|82752500 | PY06644 | Enolase | Vacuolar fusion and/or protein sorting. Deficiency in yeast leads to fragmented vacuoles and mis-sorting of vacuolar proteins |
| gi\|82540711 | PY04367 * | Small GTP-binding protein | Homologue of small GTP-binding protein sar1 [*P. falciparum* 3D7] |
| gi\|82793187 | PY07141 * | Small GTPase Rab11 related | Recycling endosomes, displays a vesicular-like structure in *P. falciparum* |
| gi\|82595062 | PY05254 * | Putative Rab2 GTPase | Ortholog of yeast SpYpt4, whose gene inactivation leads to an increase in yeast vacuole size |
| gi\|83273805 | PY01759 | Hypothetical protein | PY01759, *P. yoelii* homolog of MAL13P1.308 which is homologue of Vac8 |
| gi\|82753607 | PY06981 * | Heat shock protein hsp70 | Clathrin mediated vesicular transport predicted |
| gi\|82541361 | PY04604 * | Putative GTPase | Rab GTPase 1b [*P.falciparum*, PbANKA, Pc] |
| gi\|82541272 | PY04572 * | ADP-ribosylation factor | Regulator of vesicular traffic |
| gi\|3676478 | PY03828 | ADP-ribosylation factor GTPase-activating protein | Regulator of vesicular traffic |
| gi\|82540016 | PY04102 | rabGDI protein | Vesicular traffic |
| gi\|82596078 | PY05628 | ATPase, AAA family | *P. yoelii* homologue of PFC0140c, N-ethylmaleimide-sensitive fusion protein, putative |
| gi\|68075835 | PBANKA_  140170 | synaptobrevin-like protein **^§^** | Vesicular transport [*PbANKA, P. chabaudi*] |
| gi\|124512632 | PF08_0110 | PfRab18, GTPase **^§^** | Vesicular traffic [*Pfalciparum*] |
| *Proteasomal proteins* | | | |
| gi\|82596054 | PY00657 | 26s protease regulatory subunit 6a [Plasmodium yoelii yoelii str. 17XNL] |  |
| gi\|83314741 | PY02590 | 26s protease regulatory subunit s10b [Plasmodium yoelii yoelii str. 17XNL] |  |
| gi\|82593910 | PY00544 | 26S proteasome ATPase [Plasmodium yoelii yoelii str. 17XNL] |  |
| gi\|82594499 | PY05051 | 26s proteasome regulatory subunit s12 [Plasmodium yoelii yoelii str. 17XNL] |  |
| gi\|82915518 | PY00120 | 26S proteasome subunit [Plasmodium yoelii yoelii str. 17XNL] |  |
| gi\|82704989 | PY00768 | 26S proteasome subunit 4 protein [Plasmodium yoelii yoelii str. 17XNL] |  |
| gi\|82541772 | PY00527 | 26S proteasome subunit P40.5 [Plasmodium yoelii yoelii str. 17XNL] |  |
| gi\|83315300 | PY02805 | 26S proteasome subunit P45 [Plasmodium yoelii yoelii str. 17XNL] |  |
| gi\|82704967 | PY06176 | hypothetical protein [Plasmodium yoelii yoelii str. 17XNL] | beta3 proteasome subunit, putative [Plasmodium falciparum 3D7] |
| gi\|82914775 | PY01123 | hypothetical protein [Plasmodium yoelii yoelii str. 17XNL] | proteasome 26S regulatory subunit [Plasmodium berghei strain ANKA] |
| gi\|82915516 | PY00119 | hypothetical protein [Plasmodium yoelii yoelii str. 17XNL] | proteosome subunit, putative [Plasmodium falciparum 3D7] |
| gi\|83314707 | PY00248 | hypothetical protein [Plasmodium yoelii yoelii str. 17XNL] | 26S proteasome subunit [Plasmodium chabaudi chabaudi] |
| gi\|83316018 | PY03078 | Hypothetical protein [Plasmodium yoelii yoelii str. 17XNL] | proteasome regulatory subunit, putative [Plasmodium falciparum 3D7] |
| gi\|82540049 | PY04117 | proteasome activator pa28 subunit beta [Plasmodium yoelii yoelii str. 17XNL] |  |
| gi\|83315915 | PY03034 | proteasome subunit alpha type 2 [Plasmodium yoelii yoelii str. 17XNL] |  |
| gi\|82705582 | PY00806 | proteasome subunit beta [Plasmodium yoelii yoelii str. 17XNL] |  |
| gi\|82752562 | PY06665 | proteasome subunit beta type 2 [Plasmodium yoelii yoelii str. 17XNL] |  |

___________________________________________________________________________

Proteins that are likely to be vacuole associated ones (by analogy with yeast) are listed in three classes: (i) includes vacuole resident proteins (e.g. FV proteases and membrane transporters); (ii) involved in vesicular trafficking and (iii) includes proteasomal proteins, homologs of which have been reported to be associated with yeast vacuoles ([Kleijnen, Kirkpatrick et al. 2007](#_ENREF_1))

§ These protein were identified as hits against other *Plasmodium spp*. Homologous proteins with matching sequence could be found in *P.yoelii* genome.

***** Indicates the proteins whose homologs were identified in a study on food vacuole proteome of *P. falciparum* ([Lamarque, Tastet et al. 2008](#_ENREF_2)).
